# Microbial competition for phosphorus limits the CO_2_ response of a mature forest

**DOI:** 10.1038/s41586-024-07491-0

**Published:** 2024-06-05

**Authors:** Mingkai Jiang, Kristine Y. Crous, Yolima Carrillo, Catriona A. Macdonald, Ian C. Anderson, Matthias M. Boer, Mark Farrell, Andrew N. Gherlenda, Laura Castañeda-Gómez, Shun Hasegawa, Klaus Jarosch, Paul J. Milham, Rául Ochoa-Hueso, Varsha Pathare, Johanna Pihlblad, Juan Piñeiro, Jeff R. Powell, Sally A. Power, Peter B. Reich, Markus Riegler, Sönke Zaehle, Benjamin Smith, Belinda E. Medlyn, David S. Ellsworth

**Affiliations:** 1 College of Life Sciences, Zhejiang University, Hangzhou, China; 2 Hawkesbury Institute for the Environment, Western Sydney University, Penrith, New South Wales Australia; 3 CSIRO Agriculture and Food, Glen Osmond, South Australia Australia; 4 SouthPole Environmental Services, Zurich, Switzerland; 5 Department of Forest and Climate, Norwegian Institute of Bioeconomy Research (NIBIO), Ås, Norway; 6 Institute of Geography, University of Bern, Bern, Switzerland; 7 Agroecology and Environment, Agroscope, Zurich-Reckenholz, Switzerland; 8 Department of Biology, IVAGRO, University of Cádiz, Cádiz, Spain; 9 Department of Terrestrial Ecology, Netherlands Institute of Ecology (NIOO-KNAW), Wageningen, the Netherlands; 10 Institute of Genomic Biology, University of Illinois at Urbana-Champaign, Urbana-Champaign, IL USA; 11 Birmingham Institute for Forest Research, University of Birmingham, Edgbaston, UK; 12 School of Geography, University of Birmingham, Birmingham, UK; 13 ETSI Montes, Forestal y del Medio Natural, Universidad Politécnica de Madrid, Ciudad Universitaria, Madrid, Spain; 14 Department of Forest Resources, University of Minnesota, St Paul, MN USA; 15 Institute for Global Change Biology, University of Michigan, Ann Arbor, MI USA; 16 School for the Environment and Sustainability, University of Michigan, Ann Arbor, MI USA; 17 Max Planck Institute for Biogeochemistry, Jena, Germany

**Keywords:** Ecosystem ecology, Climate-change ecology, Forest ecology, Plant ecology, Element cycles

## Abstract

The capacity for terrestrial ecosystems to sequester additional carbon (C) with rising CO_2_ concentrations depends on soil nutrient availability^1,2^. Previous evidence suggested that mature forests growing on phosphorus (P)-deprived soils had limited capacity to sequester extra biomass under elevated CO_2_ (refs. ^3–6^), but uncertainty about ecosystem P cycling and its CO_2_ response represents a crucial bottleneck for mechanistic prediction of the land C sink under climate change^7^. Here, by compiling the first comprehensive P budget for a P-limited mature forest exposed to elevated CO_2_, we show a high likelihood that P captured by soil microorganisms constrains ecosystem P recycling and availability for plant uptake. Trees used P efficiently, but microbial pre-emption of mineralized soil P seemed to limit the capacity of trees for increased P uptake and assimilation under elevated CO_2_ and, therefore, their capacity to sequester extra C. Plant strategies to stimulate microbial P cycling and plant P uptake, such as increasing rhizosphere C release to soil, will probably be necessary for P-limited forests to increase C capture into new biomass. Our results identify the key mechanisms by which P availability limits CO_2_ fertilization of tree growth and will guide the development of Earth system models to predict future long-term C storage.

## Abstract (web summary)

Microbial pre-emption of mineralized soil P limits the capacity of trees for increased P uptake and assimilation under elevated CO_2_ and therefore restricts their capacity to sequester extra C.

## Main

Phosphorus is an essential macronutrient underpinning all life on Earth^8^. P deficiency often limits plant metabolism and growth^9^, thereby imposing a crucial potential constraint on the capacity for terrestrial ecosystems to assimilate additional C under increasing atmospheric CO_2_ concentrations^1,2^. The classic theory of pedogenesis indicates that soil P availability declines over geological timescales due to weathering^10^. Similarly, theories of natural succession posit that long-term ecosystem development can concentrate a great proportion of the available P into the slow-turnover pools such as wood and soil organic matter^11^, resulting in a decreasing proportion of P being actively recycled within the ecosystem. Thus, vegetation productivity tends to decline as natural ecosystems age^12,13^. Furthermore, as atmospheric nitrogen (N) deposition continues to augment soil N loading, ecosystems originally subject to N limitation may progressively become more limited by P availability^14^. Thus, P limitation is widespread^15,16^, and is estimated to affect one-third to half of all terrestrial vegetation^17^, including many tropical and subtropical forests, as well as woodlands of typically ancient soils of Australia^15,16,18^. Additional C uptake by trees in forests around the world dominates the global land C sink^19^, with CO_2_ fertilization suspected to be the major driver^20^, but there is still large uncertainty about future constraints on additional C sequestration imposed by limited soil nutrient availability^17,20^. In particular, few studies have directly addressed the role of ecosystem P cycling as a control on extra C assimilation and growth under future levels of atmospheric CO_2_ for forests representative of P-depleted landscapes of the tropics and subtropics.

Ecosystem models that incorporate P-cycle processes have generally predicted lower CO_2_ fertilization effects on forest growth under P limitation^7^, consistent with the findings of manipulative experiments with potted seedlings that low P availability attenuates plant responses to elevated CO_2_ (eCO_2_)^21^. Plants may have some plasticity to become more efficient in using P to support growth, or more effective in acquiring P to allow extra C sequestration in their biomass under eCO_2_ conditions^21^. However, plants may converge towards more conservative P-use strategies (such as high nutrient-resorption efficiency) as P limitation increases over time^22,23^. Thus, for natural forests subject to long-term soil development and succession, a key question is the degree to which plant plasticity may accommodate future eCO_2_-induced increases in plant nutrient demand^24^. Adequately addressing this question requires direct field-based evidence of ecosystem cycling and vegetation uptake of P by such forest systems under elevated CO_2_.

**Extended Data Fig. 1:**
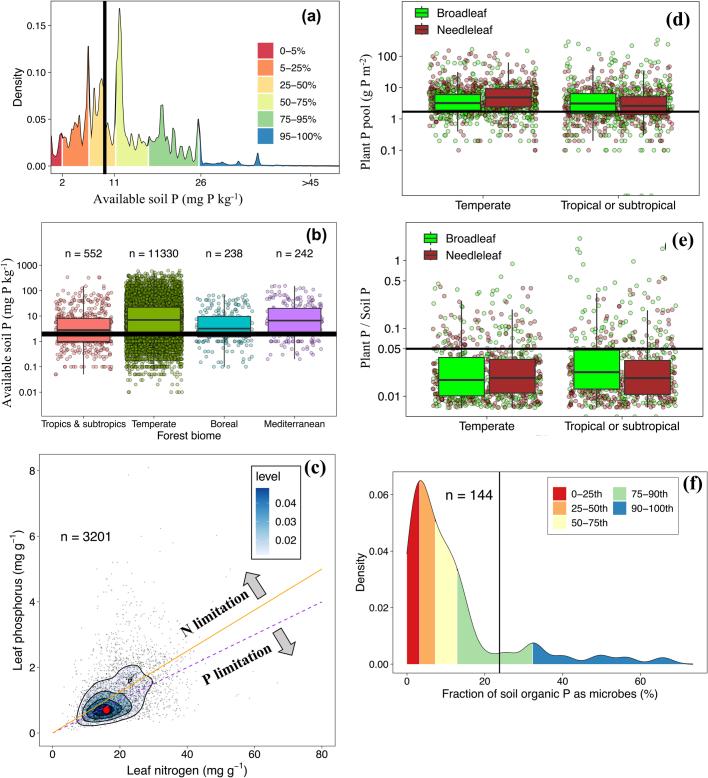
Global comparison of the phosphorus status at EucFACE. **(a)** Density distribution of global gridded plant-available soil phosphorus (P) concentration for the top 9.1 cm of the soil (mg P kg^−1^), estimated based on the Bray I method^56^. The black line indicates gridded plant-available soil P concentration for the EucFACE location (9.34 mg P kg^−1^ soil, equivalent to the ~40^th^ percentile). **(b)** Global measurements of plant-available soil P concentration (mg P kg^−1^), extracted based on the Bray I method^57^, grouped into different forest biomes. **(c)** Density plot for the paired leaf nitrogen (N) and P concentration for trees, as in ref. ^21^ but using data from ref. ^58^. Black dots indicate individual data points; red dot indicates leaf nutrient measurement at EucFACE. Orange solid and purple dotted lines indicate leaf P:N ratio of 1:16 and 1:20, respectively; see refs. ^3,21^. **(d)** Comparison of the size of the plant phosphorus (P) pool (g P m^−2^) at EucFACE to a those compiled for temperate and subtropical forest biome based on a regional dataset (n = 1969)^59^. **(e)** Same as d, but for the ratio between plant P to total soil P pool (0 − 60 cm). **(f)** Comparison of soil microbial phosphorus (P) at EucFACE to a global dataset^31^, expressed as the fraction of soil organic P as microbes (i.e., microbial P over total soil organic P over the top 60 cm of soils). The bounds of the whisker box indicate the upper quartile and lower quartile of the data, respectively, and the line within the box indicates the median. The black line indicates average value based on ambient CO_2_ plots at EucFACE (n = 3).

The limited available evidence suggests that mature trees in non-aggrading (that is, steady-state or degrading) forests may not grow faster under eCO_2_ (refs. ^3–6^), with P limitation providing a possible explanation^3,25^. Data from the *Eucalyptus* Free Air CO_2_ Enrichment (EucFACE) experiment, an evergreen mature forest growing on low-P soils (Extended Data Fig. 1), showed increased photosynthesis but no additional tree growth in the first 4 years of eCO_2_ exposure^3,5^. Concurrently, it was found that eCO_2_ did not significantly alter canopy leaf and stem P resorption or C:P stoichiometry^26^, whereas eCO_2_ increased P concentrations in the fine roots^27^. The additional C uptake through photosynthesis in turn led to a possible enhanced belowground C allocation through exudates^5^. A possible interpretation of the elevated root exudate activity is that it is part of the plant’s strategy to stimulate soil microbial activity^28,29^ and, indeed, it was associated with an ephemeral increase in net mineralization of P^30^. However, it was not clear whether this potential exchange of plant C for nutrients led to additional plant P uptake, which would potentially provide a route towards enhanced long-term C sequestration under eCO_2_. A crucial knowledge gap therefore emerged regarding how different ecosystem components interact to constrain the rate of P cycling, plant P uptake and growth response to eCO_2_.

**Fig. 1:**
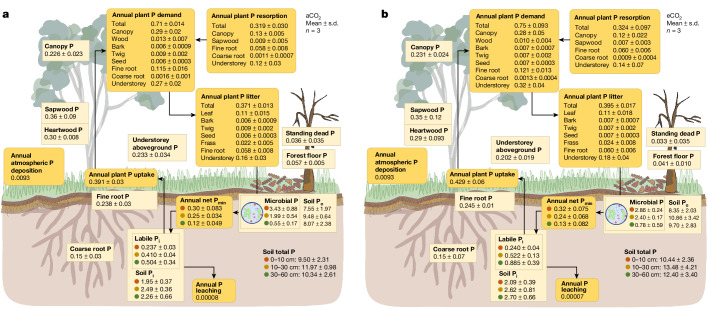
The ecosystem P budget. The ecosystem P budget under ambient CO_2_ (aCO_2_) (**a**) and eCO_2_ (**b**) treatment, assembled from data collected at EucFACE over 6 years (2013–2018). The light yellow boxes (with square corners) indicate pools (g P per m^2^), and the dark yellow boxes (with rounded corners) indicate fluxes (g P per m^2^ per year). P_i_, inorganic P; P_o_, organic P; P_min_, the net P mineralization flux. Annual plant P demand indicates the amount of P needed to support annual biomass production for the respective plant component, and this demand was met by annual plant P resorption and annual plant P uptake. For soil variables with multiple rows, the values indicate data summed over soil depths of 0–10 cm, 10–30 cm and 30–60 cm. Data are treatment mean ± s.d. *n* = 3.

**Extended Data Fig. 2:**
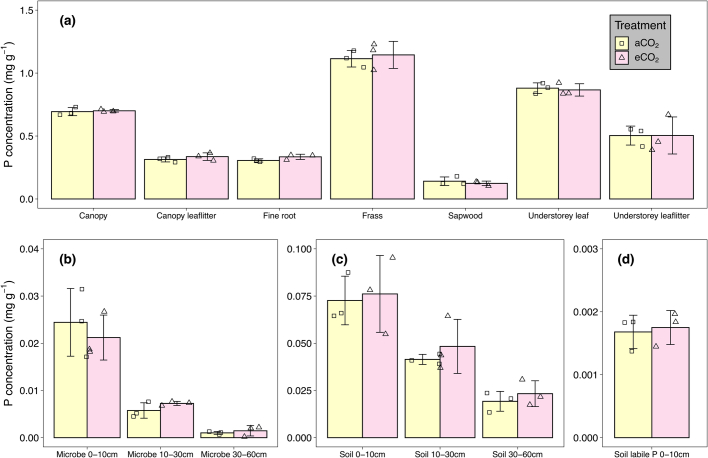
Phosphorus (P) concentration (mg g^−1^) of major variables collected at EucFACE under ambient and elevated CO_2_ treatment. **(a)** Plant tissue P concentration, including canopy leaf, canopy leaf litter, fine root, frass, sapwood, understorey leaf and understorey leaf litter P concentration; **(b)** Microbial P concentration for the soil depths of 0–10 cm, 10–30 cm, 30–60 cm; **(c)** soil total P concentration for the depths of 0−10 cm, 10–30 cm, 30–60 cm; and **(d)** labile soil inorganic P (Bray-P) for the depth of 0–10 cm. Note the different P concentration ranges in the y-axis among panels. Values are time-averaged treatment means ± standard deviations (n = 3), and open squared and triangle dots denote plot-level data under ambient and elevated CO_2_ treatment, respectively. Linear mixed-effects model indicates a lack of main CO_2_ effect (P < 0.05, Type II Wald F tests with Kenward-Roger degree of freedom)) on any individual P concentration variables (Supplementary Information 2.1).

**Extended Data Fig. 3:**
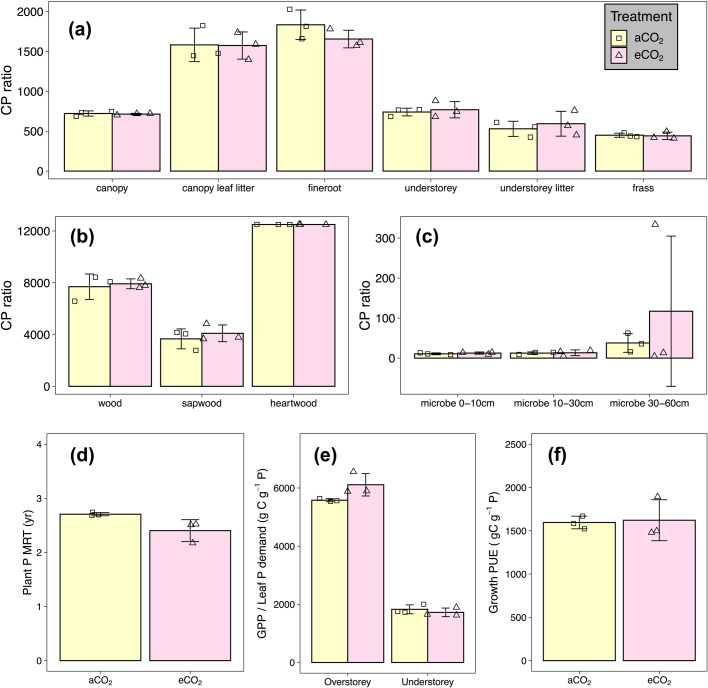
Carbon to phosphorus (C:P) ratio of major variables and plant phosphorus (P) use variables under ambient and elevated CO_2_ treatment. Variables include: **(a)** canopy leaf, canopy leaf litter, fine root, understorey aboveground, understorey litter and frass C:P ratios; **(b)** wood, sapwood and heartwood C:P ratios; **(c)** microbial C:P ratios in the 0–10 cm, 10–30 cm and 30–60 cm depths; **(d)** mean residence time (MRT) of P in plants, calculated as plant P pool over plant P uptake; **(e)** P-use efficiency to support carbon uptake of overstorey and understorey vegetation, calculated as gross primary production over leaf P demand of the respective vegetation class; and **(f)** P-use efficiency to support plant growth (overstorey and understorey combined), calculated as net primary production over plant P uptake, under ambient and elevated CO_2_ treatment. Values are time-averaged treatment means ± standard deviations (n = 3), and open squared and triangle dots denote plot-level data under ambient and elevated CO_2_ treatment, respectively.

A comprehensive assessment of the ecosystem P cycle encompassing its key biological components and biogeochemical compartments can shed light on this question. Here we present an ecosystem-scale P budget for EucFACE based on data collected over the first 6 years of CO_2_ enrichment (2013–2018; Fig. 1). The EucFACE ecosystem may be considered to be broadly representative of P-limited forests globally in terms of plant-available soil P concentrations, leaf nutrient concentrations, and the sizes of P pools in plants and soils (Extended Data Fig. 1 and Supplementary Information 1). The results from this experiment may therefore provide important insights into the functioning of forests globally. Our P budget covers all major components of the ecosystem, including concentrations (Extended Data Fig. 2), pools and fluxes connecting overstorey trees, understorey grasses, soil microorganisms, and soil organic and inorganic matter (Fig. 1), as well as associated C:P ratios (Extended Data Fig. 3). With the assembled P budget and the previous experimental evidence gathered from EucFACE^5,26,27,30^ and elsewhere^21^, we tested the following working hypotheses: (1) a large proportion of P would be sequestered in the slow-turnover woody and soil organic matter pools due to long-term ecosystem development and succession^11^, whereas only a small fraction of P in the ecosystem would be recycled to meet the annual plant nutrient demand; and (2) the additional belowground C investment under eCO_2_ (ref. ^5^) would enhance soil P availability and therefore stimulate extra plant P uptake.

## A comprehensive forest ecosystem P budget

**Fig. 2:**
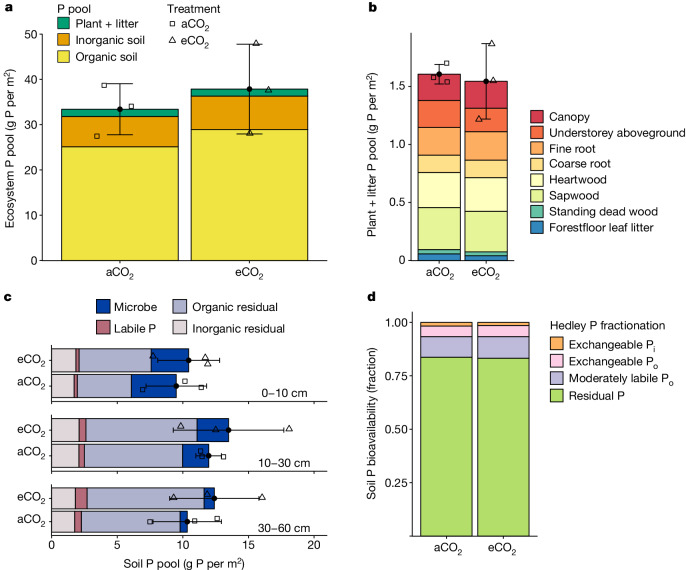
Comparison of the time-averaged ecosystem phosphorus pools (g P per m^2^) under aCO_2_ and eCO_2_ treatment. **a**, The ecosystem total P pool, split into organic soil, inorganic soil, and plant and litter. **b**, The plant and litter P pool, including canopy leaf, sapwood, heartwood, fine-root, coarse-root, understorey aboveground, forest floor leaf litter and standing dead wood pools. **c**, The soil P pool in the 0–10, 10–30 and 30–60 cm layers of the soil, split into microbial P, organic residual P (total organic minus microbial P), labile P and inorganic residual P pools (total inorganic minus labile P pool). **d**, Operationally defined fractions of soil P bioavailability, based on the Hedley fractionation method, namely, exchangeable inorganic P, exchangeable organic P, moderately labile organic P and residual P that is the remaining of total soil P, using soils over the top 10 cm depth. For **a**–**c**, the filled circles and error bars show the treatment mean ± s.d. (*n* = 3), and the open squares and triangles denote plot-level data under aCO_2_ and eCO_2_ treatment, respectively. Linear mixed-effect models show no statistically significant main CO_2_ effect (*P* < 0.05, type II Wald *F* tests with Kenward–Roger d.f.) on any individual P pool (Supplementary Information 2.1).

**Fig. 3:**
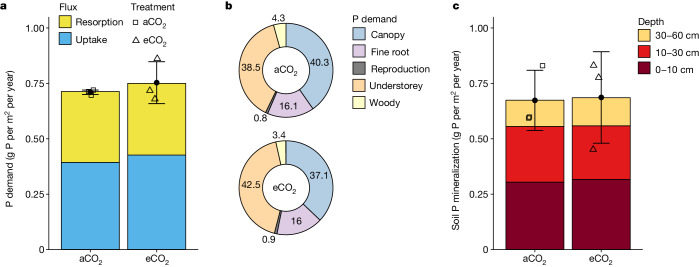
Comparison of the time-averaged aggregated ecosystem phosphorus fluxes under aCO_2_ and eCO_2_ treatment. **a**, Plant P demand flux, including plant P resorption and uptake fluxes, represents the total flux needed to support annual plant biomass production. **b**, Fractions of plant P demand, allocated into canopy, fine roots, understorey, woody and reproduction P production fluxes, with the woody component including wood, bark, twig and coarse root. **c**, The soil net P mineralization flux, including contributions from depths of 0–10 cm, 10–30 cm and 30–60 cm. The filled circle dots and error bars show the treatment mean ± s.d. (*n* = 3), and the open square and triangle dots denote plot-level data under aCO_2_ and eCO_2_ treatment, respectively. Linear mixed-effect models show no statistically significant main CO_2_ effect (*P* < 0.05, type II Wald *F* tests with Kenward–Roger d.f.) on any individual P flux (Supplementary Information 2.1).

Our P budget provides direct field-based evidence to support hypothesis 1 that a large proportion of P was sequestered in the slow-turnover live woody and soil organic matter pools (soil P pool of 31.8 ± 5.7 g P per m^2^ for the top 60 cm depth versus plant P pool of 1.60 ± 0.08 g P per m^2^; mean ± s.d. of ambient plots; *n* = 3; Fig. 2a,b), whereas only a small fraction of P was recycled in the ecosystem to support annual plant nutrient demand (0.71 ± 0.01 g P per m^2^ per year; Fig. 3a).

In soils, most of the P was present in organic rather than inorganic pools (25.1 ± 4.8 and 6.7 ± 1.16 g P per m^2^, respectively; Fig. 2a). Soil microorganisms contained a sizable amount of P (5.97 ± 1.43 g P per m^2^; Fig. 2c), representing 24% of the soil organic P pool, which is at the top end of such values from a global dataset^31^ (median, 7.2%; mean, 11.6%; Extended Data Fig. 1 and Supplementary Information 1). The sharp contrast between plant and microbial P pools (that is, >3.5× larger microbial P pool compared to the plant P pool) indicates a competitive imbalance for the labile soil inorganic P pool^13^. In fact, only about 3% of soil P was readily extractable and therefore directly available for plant uptake (1.15 ± 0.28 g P per m^2^; Fig. 2c); this small fraction of bioavailable P was independently supported by the Hedley fractionation estimate for this site^32^ (around 2%) (Fig. 2d).

In plant and litter pools, the slow-turnover woody components contained 53% of the total P pool (that is, 0.36 ± 0.09, 0.30 ± 0.01, 0.15 ± 0.03 and 0.04 ± 0.04 g P per m^2^ in sapwood, heartwood, coarse root and standing dead wood pools, respectively; Fig. 2c). An additional 4% was present on the forest floor as litter (that is, 0.06 ± 0.005 g P per m^2^; Fig. 2c). The remaining 43% of the total plant and litter P was present in the fast-turnover pools, approximately equally split into canopy tree leaves, understorey shoots and fine roots (that is, 0.23 ± 0.02, 0.23 ± 0.04 and 0.24 ± 0.03 g P per m^2^, respectively; Fig. 2b).

The P cycling in this forest was mainly driven by the annual turnover of the plant pools (Fig. 3a,b), with overstorey leaf production and understorey aboveground biomass production dominating the total plant P demand (both around 40%; Fig. 3b). A sizable proportion of the canopy P (14%) was consumed and deposited as frass by leaf-chewing insect herbivores, estimated at 0.04 ± 0.009 g P per m^2^ per year. Total plant P resorption had an important role in meeting the annual plant nutrient demand (45%; 0.32 ± 0.03 g P per m^2^ per year; Fig. 3a), with overstory trees being more efficient at resorbing P than understory grasses (Supplementary Information 1). The resorption fraction for canopy leaves (55%) was slightly above the global average (48%) reported for evergreen broadleaf forests^12^, suggesting an efficient use of P by trees at EucFACE. The remaining P demand was met by plant P uptake, estimated to be 0.39 ± 0.03 g m^−2^ yr^−1^ (Fig. 3a). This flux was considerably lower than the net P mineralization flux estimated for the top 60 cm of the soil column (0.67 ± 0.14 g m^−2^ yr^−1^; Fig. 3c), suggesting that the soil P supply was sufficient to meet the annual plant P demand. Nevertheless, given that 92% of the fine-root and similar fractions of microbial biomass and microbial P content were found in the top 30 cm of the soil^33^, it is probable that plant P uptake occurred predominantly in the shallower soil layers. Fluxes for soil P leaching and atmospheric P deposition were negligible at the ecosystem scale (Fig. 1), suggesting an essentially closed P cycle in this forest, which also means that the internal recycling of P is essential to support plant growth and metabolism in the EucFACE ecosystem.

## P-cycle responses to eCO_2_

**Fig. 4:**
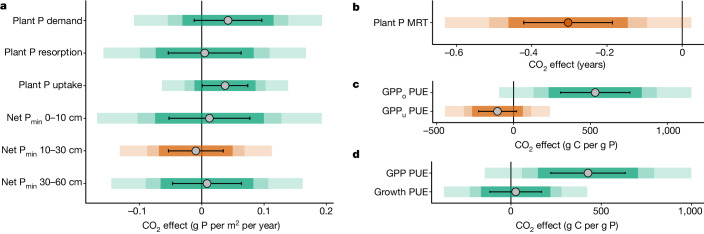
The time-averaged CO_2_ effect on major phosphorus cycle variables. **a**, The CO_2_ effect (g P per m^2^ per year) on major plant and soil P fluxes, namely the CO_2_ effect on plant P demand, plant P resorption and plant P uptake, and net soil P mineralization fluxes in the 0–10 cm, 10–30 cm and 30–60 cm depths. **b**, The CO_2_ effect (in years) on the mean residence time (MRT) of P in plants. **c**, The CO_2_ effect (g C per g P) on P-use efficiency to support overstorey and understorey gross primary production (GPP_o_ and GPP_u_, respectively), calculated as GPP_o_ and GPP_u_ over the respective leaf P production fluxes. **d**, The CO_2_ effect (g C per g P) on P-use efficiency to support total GPP and plant growth, with the latter calculated as the total plant net primary production of overstorey and understorey combined over plant P uptake flux. For **a**–**d**, the circles indicate the mean absolute CO_2_ effect, calculated by using elevated minus ambient CO_2_ treatment (*n* = 3), and the coloured bars indicate the confidence intervals at 95%, 85% and 75% (two-tailed *t*-tests; the lighter colours indicate higher confidence levels). If the coloured bars intercept with zero, it means that the reported CO_2_ effect size is not significantly different from zero at the respective confidence levels. The error bars indicate s.e.m. values of the treatment means.

**Extended Data Fig. 4:**
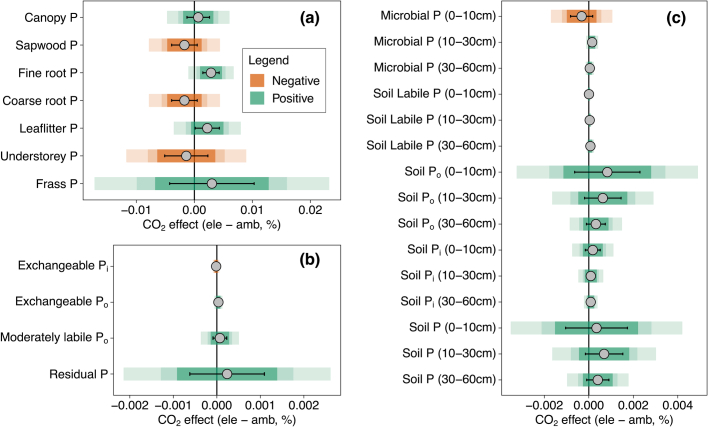
Time-averaged CO_2_ response of all individual phosphorus (P) concentration variables. **(a)** CO_2_ effect on major vegetation P concentration (%); **(b)** CO_2_ effect on major soil Hedley fractionation P concentrations (%), and **(c)** CO_2_ effect on major soil P concentration (%). The solid dots indicate the mean absolute CO_2_ effect, calculated by using elevated minus ambient CO_2_ treatment (n = 3), with the coloured bars indicating confidence intervals at 95%, 85% and 75% (two-tailed t-test, with shallower colours indicating higher confidence levels). If the coloured bars intercept with zero, it means that the reported CO_2_ effect size is not significantly different from zero at the respective confidence levels. The black bars indicate standard errors of the treatment difference.

**Extended Data Fig. 5:**
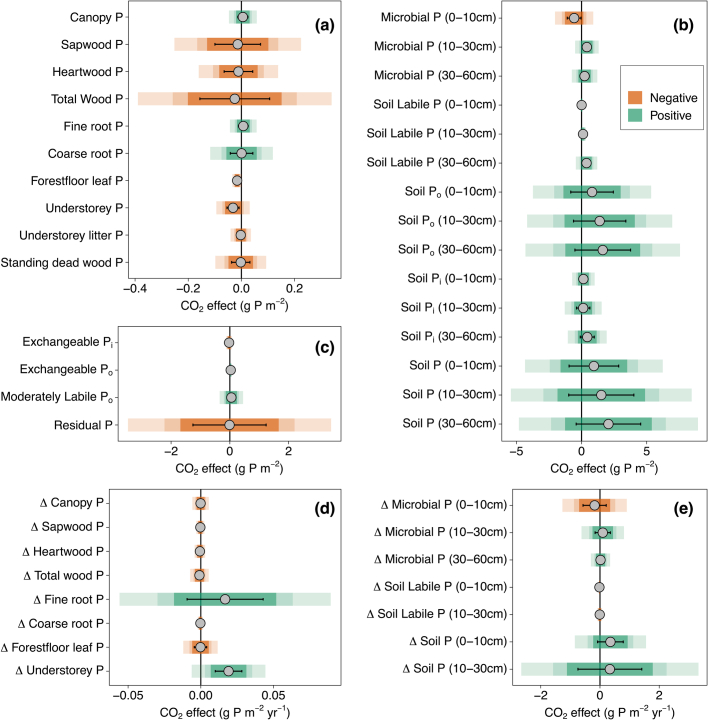
Time-averaged absolute difference between ambient and elevated CO_2_ plots on major ecosystem phosphorus (P) pools and annual incremental change in major ecosystem P pools. **(a)** The CO_2_ effects on plant P pools, including canopy, sapwood, heartwood, total wood, fine root, coarse root, forest floor leaf litter, understorey, understorey litter, and standing dead wood P pools; **(b)** the CO_2_ effects on soil P pools, including microbes, labile P, soil organic, soil inorganic, and total soil P pools in the 0–10 cm, 10–30 cm, and 30–60 cm depths, respectively; **(c)** the CO_2_ effect on soil P pools in the top 10 cm of the soil, classified based on the Hedley fractionation method, including exchangeable inorganic P (P_i_), exchangeable organic P (P_o_), moderately labile P_o_, and occluded P, which is the remaining P that we consider to be relatively unavailable to plants; **(d)** the CO_2_ effects on annual incremental change in major plant P pools, including canopy, sapwood, heartwood, total wood, fine root, coarse root, forest floor leaf litter and understorey P pools; and **(e)** the CO_2_ effects on annual incremental change in major soil P pools, including microbes in the three depths profile, and labile and total soil P pools in the 0–10 cm and 10–30 cm depths. The shorter lists in **(d)** and **(e)** reflect the lack of data to calculate annual incremental changes in depth. The solid dots indicate the mean absolute CO_2_ effect, calculated by using elevated minus ambient CO_2_ treatment (n = 3), with the coloured bars indicating confidence intervals at 95%, 85% and 75% (two-tailed t-test, with shallower colours indicating higher confidence levels). If the coloured bars intercept with zero, it means that the reported CO_2_ effect size is not significantly different from zero at the respective confidence levels. The black bars indicate standard errors of the treatment difference.

Averaged among the experimental treatment plots (that is, FACE rings), most of the P-related variables did not exhibit significant eCO_2_ responses at the 95% confidence level and the effect sizes were generally quite modest (Fig. 4, Extended Data Figs. 4 and 5 and Supplementary Information 2.1); this result does not support hypothesis 2 that additional belowground C investment would increase soil P availability and plant P uptake under eCO_2_. The evidence for the differences in the budget numbers between control and eCO_2_ treatment was statistically weak, reflecting a low sample size relative to the inherent variability in the field—a common drawback of FACE experiments. Nonetheless, this comprehensive P budget, taken as a whole, is still useful in that it provides a cohesive and systematic framework to examine the relative responses of different P-cycle components to altered CO_2_ concentration. Here we used this budget to interpret the eCO_2_ responses (Fig. 4 and Extended Data Figs. 4 and 5).

Our results show very weak evidence that the mean plant P demand to support annual production of plant biomass (overstorey and understorey combined) was higher under eCO_2_ (+6% or +0.043 ± 0.055 g P per m^2^ per year, mean ± s.e.m. of the treatment difference; Fig. 4a). This effect may reflect the increased biomass production in the understorey^5^ and the increased P concentration in the fine roots with eCO_2_ (ref. ^27^) (Extended Data Fig. 4), and is unlikely to be met by plant P resorption response to eCO_2_ (+1% or +0.003 ± 0.06 g P per m^2^ per year; Fig. 4a and Extended Data Fig. 5). Changes in understorey species composition^34^ may have played a role in the observed changes of fine-root P concentration with eCO_2_ (ref. ^27^). Plant P uptake also showed weak evidence of a modest positive eCO_2_ response (+8% or +0.033 ± 0.036 g P per m^2^ per year; Fig. 4a). Comparing plant P uptake and plant P demand responses to eCO_2_ suggests that additional plant P uptake would have a dominant role in meeting the extra demand if there was a detectable increase in plant P demand with eCO_2_. Furthermore, there was strong evidence that the mean residence time (MRT) of P in plants was lower in eCO_2_ plots (−11% or −0.3 ± 0.12 years; Fig. 4b). This significant difference suggests a faster plant P cycling in eCO_2_ plots; thus, the modest increase in plant P uptake with eCO_2_ is possibly biologically important relative to the size of plant P pool. Similarly, plants, and particularly overstorey trees, have increased P-use efficiency in leaves to support C uptake with eCO_2_ (moderate evidence; +10% or +531 ± 225 g C per g P; Fig. 4c). However, this did not lead to a more efficient use of P to support overall plant growth (+2% or +26 ± 143 g C per g P; Fig. 4d and Extended Data Fig. 3). This result suggests that plant growth responses to eCO_2_ are probably proportional to the corresponding plant P uptake response, meaning that extra growth with eCO_2_ would only be possible through additional plant P uptake.

Nevertheless, there was little to no evidence for eCO_2_-induced responses of plant P uptake, net P mineralization (+0.013 ± 0.143 g P per m^2^ per year; Fig. 4), soil labile P concentration (Extended Data Fig. 5) or soil phosphatase enzyme activity^35^, despite the increased belowground C allocation^5^. The large microbial P pool (Fig. 2) and the sharp contrast between the amount of P stored in microorganisms and those actively recycled in the ecosystem to support annual plant production (Fig. 3) suggests that microbial competition for P is strong. The annual incremental change in the microbial P pool did not exhibit any detectable eCO_2_ response (−0.067 ± 0.71 g P per m^2^ per year; Extended Data Fig. 5), but any change in this quantity in response to eCO_2_ would be small in absolute terms relative to the large total microbial P pool. Taken together, we infer that microbial competition for P may constrain the rate of soil P supply to plants by pre-emptive exploitation of the mineralized P, limiting the amount of soluble P remaining for plants and therefore precluding plant growth response to eCO_2_.

## Microorganisms limit plant eCO_2_ responses

By constructing a comprehensive ecosystem P budget, we provide direct field-based evidence of how P, as a limiting macronutrient, is distributed through the plant–microorganism–soil continuum in a P-poor mature forest ecosystem, and how P availability constrains ecosystem productivity and its response to eCO_2_. In particular, soil microorganisms had amassed a large proportion of the soil P and displayed limited flexibility to respond to an eCO_2_-induced increase in belowground C investment from plants, thereby limiting the rate of plant-available soil P supply in response to eCO_2_. Notably, although we have relatively high statistical confidence with this interpretation, our results are subject to uncertainties due to the inherent spatial and temporal variability in this field-based, long-term experiment. Nevertheless, with the effect sizes and the confidence intervals reported, this first comprehensive ecosystem P budget still provides mechanistic insights into how P availability might broadly constrain ecosystem responses to eCO_2_ in low-P forest ecosystems.

The large proportion of biomass P stored in microorganisms in this forest is not unique^13,36^, and potentially reflects the advanced stage of ecosystem development^9,13^. In this respect, the mature, non-aggrading status of EucFACE differs from that of other forest FACE experiments^17^. The lack of an apparent CO_2_ effect on soil microbial biomass^5^ and P pool, despite the additional belowground C investment by plants, suggests that microorganisms are possibly conservative in releasing P in exchange for C in the low-P soils at EucFACE^37^. However, given that microbial C-use efficiency typically declines with lower soil P availability^38^, it is also possible that the eCO_2_-induced increase in belowground C allocation into the low-P soils at EucFACE was not enough to stimulate extra P mineralization, even after 6 years of CO_2_ enrichment. The lack of response to eCO_2_ in terms of the relative abundance of saprotrophic and mycorrhizal fungi in soil over the first 5 years supports this interpretation (Supplementary Information 3).

It remains to be seen whether the eCO_2_-induced increase in plant belowground C allocation leads to a more detectable response of P availability to eCO_2_ being realized over longer time frames. The observed reduction in soil pH at depth is consistent with enhanced plant exudates and provides an indication that this may occur^33^—it reflects an additional pathway through which soil P can be made available to plants under eCO_2_ (ref. ^39^). Extra plant nutrient uptake is also possible if plants invest in deeper or more extensive rooting systems under eCO_2_, enabling them to explore deeper layers of the soil, as suggested in other FACE studies^40,41^. Nevertheless, given that the likely increase in plant P demand with eCO_2_ was largely a reflection of the enhanced understorey biomass turnover^5^, understorey vegetation could be more competitive to acquire any newly available P with eCO_2_ than overstory trees. Thus, long-term enhancement of tree growth and ecosystem C storage under eCO_2_ remains questionable in this low-P forest system.

## Future modelling implications

The response of P-limited forest ecosystems to eCO_2_ is a major source of uncertainty in global land surface models^7,42,43^, but is essential knowledge to inform climate change mitigation strategies^44^. Current models generally predict that soil P availability would impose a critical constraint on the C-sequestration potential of forests globally^45,46^. However, models differ widely in their predicted CO_2_ responses, in part because they adopt competing, plausible representations of P-cycle processes, particularly regarding plant strategies for P use and acquisition^7^. Our complete assessment of the ecosystem P budget provides a rare opportunity to benchmark both the prediction accuracy and the verity of mechanisms assumed in the model simulations, especially for those concerning mature forests grown on low-P soils.

Our results disagree with the predictions of two P-enabled models from before the start of EucFACE that suggested that soil P processes have no material effect on (that is, did not constrain) plant growth response to eCO_2_ (ref. ^43^). In fact, the strong microbial constraint observed at EucFACE highlights the need to more accurately represent the C cost for nutrient acquisition, as well as the biological and biochemical processes that regulate soil P cycling responses to eCO_2_ (refs. ^47,48^). These processes are typically not well represented in land surface models^7,42^. For example, a recent multimodel intercomparison for a P-limited tropical rainforest^7^ showed that models with assumptions that upregulate plant P acquisition can effectively alleviate plant P limitation under eCO_2_ as a consequence. However, they do so through an increased desorption of the less labile soil inorganic P pool, which, in the models, does not incur any C cost—an unrealistic assumption that does not involve any identified biological processes^7^. Including a trade-off between plant C investment and nutrient acquisition in models has resulted in much lower global estimates of net primary production^49^. However, there is still the need for further data to quantitatively characterize this trade-off and the processes involved in regulating its effectiveness under eCO_2_ (refs. ^50,51^). In comparison, for models that allow upregulation of plant P-use efficiency such as through flexible plant tissue C:P stoichiometry, an initial positive biomass response to eCO_2_ is commonly predicted^7^. However, flexible stoichiometry also reduces litter quality for decomposition, thereby making nutrients increasingly unavailable to plants over time. It is therefore highly unlikely that these models will correctly simulate the observed faster plant P cycling with eCO_2_ at EucFACE. Thus, models need to impose more realistic plasticity and biological limits in plant P-use efficiency^24^. Currently, such improvements in models are limited by the availability of species-specific data on the relevant traits and their functional responses to eCO_2_ variation^21,51^.

Taken together, our results suggest that a solid understanding of C-nutrient feedbacks between plants, soils and microorganisms is critical to improve our ability to predict land C sink under climate change. Although plants, and overstorey trees in particular, were highly efficient at using P in the EucFACE mature forest ecosystem, they were not able to capture more P after 6 years of eCO_2_ exposure, despite enhanced belowground C investment. The competitive superiority of the soil microbial community, relative to vegetation, with respect to P uptake provides one probable explanation for the lack of a tree growth response to eCO_2_. Our findings for this P-limited mature forest ecosystem in Australia are probably relevant to understanding the long-term capacity of forests of the tropics and subtropics to capitalize on the production-enhancement potential of rising atmospheric CO_2_, and therefore to help maintain the persistence of the global land C sink under climate change.

## Methods

### Site description

The EucFACE experiment is located in a remnant native Cumberland Plain woodland on an ancient alluvial floodplain in western Sydney, Australia (33° 37′ S, 150° 44′ E, 30 m in elevation). The site has been unmanaged for over 90 years and is characterized by a humid temperate-subtropical transitional climate with a mean annual temperature of 17 °C and mean annual precipitation of about 800 mm (1881–2014, Bureau of Meteorology, station 067105 in Richmond, New South Wales, Australia; http://www.bom.gov.au). The soil is formed from weakly organized alluvial deposits and is primarily an Aeric Podosol with areas of Densic Podosol (Australian soil classification)^52^. The open woodland (600–1,000 trees per ha) is dominated by *Eucalyptus tereticornis* Sm. in the overstorey, while the understorey is dominated by the C_3_ grass *Microlaena stipoides* (Labill.) R.Br^5,53^, and is co-dominated by ectomycorrhizal and arbuscular mycorrhizal fungi species in soils^29,54^. Evidence from a *Eucalyptus* woodland in Southwest Australia indicates that *M. stipoides* can release phytosiderophores (that is, organic exudates with strong chelating affinity) under low-P conditions to mobilize soil P^55^. The vegetation within three randomly selected plots (~450 m^2^ each) has been exposed to an eCO_2_ treatment aiming for a CO_2_ mole fraction of 150 μmol mol^−1^ above the ambient concentration since February 2013 (ref. ^28^). The other three plots were used as control plots representing the aCO_2_ treatment, with identical infrastructure and instrumentation as the treatment plots.

An earlier study has estimated the ecosystem C budget for the site under both ambient and elevated CO_2_ treatment^5^; here we report some relevant numbers in Extended Data Table 1. Total soil N for the top 10 cm of the soil is 151 ± 32 g N per m^2^, and available soil P is 0.24 ± 0.04 g P per m^2^, broadly comparable to soils in tropical and subtropical forests globally^56,57^ (Extended Data Fig. 1). The N:P ratio of mature canopy leaves is 23.1 ± 0.4 (ref. ^26^), above the stoichiometric ratio of 20:1 to suggest likely P limitation^58^ (Extended Data Fig. 1). Plant P pool and plant P to soil P ratio at EucFACE is also comparable to those seen in other temperate or tropical forests^59^ (Extended Data Fig. 1). It has been shown that P fertilization in the same forest increases tree biomass, suggesting soil P availability is a limiting factor for plant productivity at the site^25^.

### Estimates of P pools and fluxes

We estimated plot-specific P pools and fluxes at EucFACE based on data collected over 2013–2018 (ref. ^60^). We defined pools as a P reservoir and annual increments as the annual change in the size of this reservoir. We reported all P pools in the unit of g P per m^2^ and all P fluxes in the unit of g P per m^2^ per year. For data that have subreplicates within each treatment plot, we first calculated the plot means and the associated uncertainties (for example, standard errors), and then used these statistics to calculate the treatment means and their uncertainties. For data that have repeated measurements over time, our principle is to first derive an annual number and then calculate the multiyear means and their associated uncertainties. Pools were calculated by averaging all repeated measurements within a year. For fluxes with repeated measurements within a year, we calculated the annual totals considering the duration over which the flux was measured. Below, we report how individual P pools and fluxes were estimated in detail.

#### Plant P pools

The total standing plant P pool was estimated as the sum of all vegetation P pools, namely: canopy, stem, fine-root, coarse-root, understorey aboveground, standing dead wood and forest floor leaf litter P pools. We generally adopted a concentration by biomass approach to estimate the plot-specific plant P pools unless otherwise stated in the methods below.

Fully expanded green mature leaves from the overstorey trees were collected from 3–4 dominant or co-dominant trees per plot in February, May and October between 2013 and 2018, whereas senesced leaves were collected from 2–3 litter traps (~0.2 m^2^) per plot in each February between 2013 and 2018 (ref. ^26^). Green understorey leaves were collected in 2013, 2015 and 2017, and senesced understorey leaves were collected in June 2017. Total P concentrations of green and senesced leaves were determined using a standard Kjeldahl digestion procedure, using pure sulfuric acid and hydrogen peroxide (H_2_O_2_, 30%). The total P concentrations of the Kjeldahl digests were colorimetrically analysed at 880 nm after a molybdate reaction in a discrete analyzer (AQ2 Discrete Analyzer, SEAL Analytical, EPA135 method). Overstorey leaf P and understorey aboveground P pools were estimated based on the respective plot-level mean P concentration of the green leaves and the corresponding biomass data^5^. The forest-floor leaf litter P pool was estimated on the basis of the forest-floor leaf litter pool and the senesced overstorey leaf P concentration. Woody materials (that is, bark, sapwood and heartwood) were sampled in November 2015 from breast height in three dominant trees per FACE plot. Sapwood was defined as the outer 20 mm of wood beneath the bark^26,61^. All woody materials were digested using the Kjeldahl procedure and analysed for total P concentration by inductively coupled plasma optical emission spectroscopy (Perkin-Elmer). For all chemical analyses, we ran blind internal standards, using NIST Standard Reference Material 1515 (U.S. National Institute of Standards and Technology) for quality-control purposes. Sapwood and heartwood P pools were calculated using the respective P concentrations and biomass data^5^ at the plot level. The total wood P pool was estimated as the sum of the sapwood and heartwood P pools. Standing dead wood P pool was estimated on the basis of standing dead woody biomass data, which pooled all dead trees within each plot together. We assumed the same sapwood and heartwood partitioning and used the respective P concentrations to obtain the total standing dead wood P pool for each plot. Coarse-root P pool was calculated based on coarse-root biomass and sapwood P concentration, with coarse-root biomass estimated based on an allometric relationship developed for Australian forest species^62^.

The fine-root P concentration was determined on the basis of fine-root samples collected using eight intact soil cores over the top 30 cm of the soils within 4 randomly located, permanent 1 m × 1 m subplots in each FACE plot. Fine roots included roots of both overstorey and understorey vegetation, and were considered fine roots when their diameter was <2 mm and no secondary growth. The samples were collected using a soil auger (5 cm diameter) in February 2014, June 2014, September 2014, December 2014, May 2015, September 2015 and February 2016. After sorting and oven-drying, small representative subsamples (~100 mg) from each standing crop core for each date were ground on the Wig-L-Bug dental grinder (Crescent Dental Manufacturing). Total P concentration in the sample was assessed using X-ray fluorescence spectrometry (Epsilon 3XLE, PANalytical)^63^. We then used fine-root biomass data collected in December 2013 to extrapolate the depth profile in fine-root biomass down to the 30–60 cm soil horizon. We considered the intermediate root class (that is, roots with a diameter between 2–3 mm) to have the same P concentration as those of the fine root, and we pooled the two root classes into the fine-root P pool. We estimated the fine-root P pool based on fine-root P concentration and the biomass data for each plot.

#### Vegetation P fluxes

Total plant P demand was estimated as the sum of all of the vegetation P fluxes to support the annual biomass growth, namely: canopy, stem, branch, bark, twig, reproduction, fine-root, coarse root and understorey aboveground P production fluxes. Each plant P production flux was calculated by multiplying the respective P concentration measured in the live plant organ and the corresponding annual biomass production rate. Specifically, canopy leaf, branch, bark, twig and reproductive structure biomass production fluxes were estimated on the basis of the monthly litter data collected from circular fine-mesh traps (~0.2 m^2^) at eight random locations for each FACE plot^5^. We independently estimated a herbivory consumption flux of the canopy leaves and added this flux on top of the canopy leaf litter flux to obtain the total canopy leaf production flux^5,64,65^. Considering an approximately annual canopy leaf lifespan^66^, the estimated canopy leaf P production flux was slightly more than sufficient to replace the entire canopy P pool annually. The canopy P pool was a conservative estimate as it takes the mean of the time-varying canopy size, whereas the canopy leaf P production flux takes the cumulative leaf litterfall. The production fluxes of wood and coarse root were estimated based on the annual incremental change of wood and coarse-root biomass, respectively. The production flux of fine roots was estimated based on samples collected from in-growth cores at four locations per plot. The production flux of the understorey aboveground component was estimated on the basis of biomass clippings taken between 2014 and 2017, assuming one understorey turnover per harvest interval^5^. The P concentrations in green canopy and understorey leaves were used to calculate canopy and understorey aboveground P production fluxes. The sapwood P concentration was used to calculate wood and coarse-root P production fluxes. P concentrations in bark, twig, reproductive structure and branch were assumed to be the same as those in sapwood.

Plant P litter fluxes of canopy and understorey leaves were calculated using the respective litter production flux and the P concentration in senesced plant tissue. Litter P fluxes of bark, branch, twig and reproductive structure were assumed to be the same as their production P fluxes. Frass was collected monthly for 2 years from all 8 litter traps per FACE plot between late 2012 and 2014 (ref. ^64^). Frass was oven-dried at 40 °C for 72 h. A microscope was used to determine the frass of leaf-chewing herbivores using shape, texture and colour, and excluding lerps and starchy excretions by plant-sucking psyllids^67^. After sorting, frass samples were weighed, pooled by plot and ground into a fine powder for chemical analysis. Monthly P concentrations were determined by placing 50 mg of sample in a muffle furnace (550 °C) for 8 h. The resulting ash was dissolved in 5 ml of 1% perchloric acid and the total P was quantified using the ascorbic acid–molybdate reaction^68^. Frass P litter flux was estimated on the basis of the frass P concentration and the corresponding litter flux was measured from the litter traps.

The plant P-resorption flux was estimated as the sum of canopy, understory aboveground, sapwood, fine-root and coarse-root P resorption fluxes. Plant P-resorption rates for the canopy and understorey leaves were estimated on the basis of the corresponding difference between fully expanded live and senesced leaf P concentrations. The sapwood P-resorption flux was estimated as the difference in P concentrations between sapwood and heartwood, and we used the same fraction to estimate coarse-root resorption flux. The fine-root P-resorption coefficient was assumed to be a constant of 50% due to the difficulty in separating live and dead components of the fine roots^69^.

Total plant P uptake was estimated as the net difference between plant P-demand and plant P-resorption fluxes. Overstorey and understorey P-use efficiency to support the respective photosynthesis were calculated as the respective gross primary production divided by their corresponding leaf P-production flux. The plant P-use efficiency was estimated as the total plant P demand over the net primary production of both overstorey and understorey vegetation, because fine-root production includes contributions from both overstorey and understorey plants. The plant P MRT (years) was calculated as the standing vegetation P pool (excluding the heartwood and coarse root) over the plant P-uptake flux.

#### Soil P pools

Soil P pools were determined based on soil collected from four 2 m × 2 m subplots within each of the six FACE plots. A grid system was assigned to each soil subplot, and sampling locations were noted to ensure the same location was not sampled more than once. At the time of sampling, three soil cores (3 cm diameter) were collected from each sample location and pooled into one composite sample for each subplot. Pooled soils were sieved (<2 mm). Soils were repeatedly sampled over the top 10 cm between 2013 and 2015, once for the 10–30 cm depth in 2013 and once in 2017 for 0–10 cm, 10–30 cm and 30 cm to a hard clay layer located at variable depth across the site (median 56 cm, range 35–85 cm). P pools were calculated on the basis of the measured P concentrations and mean soil bulk density measures at each depth class for each FACE plot (Extended Data Table 1). The pool size for 2017 up to 60 cm depth was calculated using the concentration measured below 30 cm and to the clay layer.

In soil from 2013 to 2015, the total soil P concentration was determined on finely milled (MM 400, Retsche) oven-dried (40 °C, 48 h) soils after aqua regia digestion and inductively coupled plasma mass spectrometry (ICP-MS) analysis (Environmental Analysis Laboratory, Southern Cross University). For 2017 soils, total, organic and inorganic soil P were determined by two methods. Using an approach described previously^70^, 1 g of oven-dried (40 °C, 48 h) finely ground (MM 400, Retsche) soil was either ignited for 1 h at 550 °C (for total P) or extracted untreated (for inorganic P) for 16 h with 25 ml of 0.5 M H_2_SO_4_ and the extracts passed through a 0.2 µm filter before colorimetric analysis^71^. Organic P was determined as the difference between total P and inorganic P. As the method has been shown to overestimate organic P in highly weathered soils^72^, we also used a previously described approach^73^ whereby 2 g of milled soil was extracted for 16 h with 30 ml in a 0.25 M NaOH + 0.05 M EDTA solution. After passing the extract through a 0.2 µm filter, the filtrates were analysed for total P concentration (ICP-MS) and inorganic P using the Malachite Green method^70^ and organic P was computed as the difference between total P and inorganic P. Values obtained for total P, inorganic P and organic P that were determined using both methods were similar and values for the respective P classes were averaged across methods. Total P values determined in 2017 were also similar to those obtained previously using the aqua regia method.

To determine operationally defined soil P pools, soils collected from the top 10 cm of the soil in 2013 were sequentially extracted with 1 M NH_4_Cl, 0.5 M NaHCO_3_ (pH 8.5), 0.1 M NaOH, 1 M HCl and 0.1 M NaOH according to a modified Hedley fractionation method^74^. Each extract was analysed colorimetrically for determination of inorganic P using the Malachite Green method^70^. To determine organic P, a subsample of extracts (2.5 ml) was digested with 0.55 ml 11 M H_2_SO_4_ and 1.0 ml 50% ammonium peroxydisulfate as previously described^74^, and inorganic P determined as before. Organic P was defined as the difference in inorganic P between digested and undigested samples. The occluded P was defined as the total P (as determined by aqua regia, described above) minus the sum of all other P concentrations^75^. We used the Hedley fractionation method to discriminate soil P pools of different chemical extractability as a potential indicator of soil P bioavailability. Notably, this method may introduce artifacts in certain chemical fraction estimates^76^. We therefore took a conservative approach by grouping less-available soil P fractions as a residual P pool, and reported the more easily extractable fractions separately, which we operationally defined as exchangeable inorganic P, exchangeable organic P and moderately labile organic P.

The extractable inorganic P pool (that is, labile P_i_) was determined quarterly between 2013 and 2015 on 0–10 cm layer soils using the Bray-1 P extraction^30,73^ method, and once in 2017 (0–10 cm, 10–30 cm and 30–60 cm)^33^. Phosphate concentrations in soil extracts were determined colorimetrically using the molybdate blue assay (AQ2 Discrete Analyzer SEAL Analytical) using an established method for available P (EPA-118-A rev.5). The proportion of change in concentration across depth in 2017 was applied to the averaged 2013–2015 measurements to estimate the concentrations across 10–30 cm and 30–60 cm depths.

The microbial P pool, comprising bacteria, archaea, protozoa and fungi, was assessed within 2 days of sampling using chloroform fumigation extraction^77^, and estimated quarterly between 2014 and 2015 for 0–10 cm and once in 2017 (0–10 cm, 10–30 cm and 30–60 cm). In brief, 3.75 g soil was fumigated in the dark for 24 h. Phosphorus was extracted from fumigated and unfumigated samples using the Bray-1 P extraction method as above. Microbial biomass P was determined as the difference in extractable P between fumigated and unfumigated samples. A conversion factor of 0.4 was used to calculate the microbial P pool^77^. The proportion of change in microbial P concentration across depth measured in 2017 was applied to the averaged 2014–2015 measurements per plot (0–10 cm) to estimate the concentrations across 10–30 cm and 30–60 cm depths.

#### Soil P fluxes

The soil net P-mineralization flux (gross mineralization minus gross immobilization) was determined in situ at the 0–10 cm depth on a quarterly basis as the change in phosphate concentration between two timepoints between January 2013 and January 2016 using PVC pipes^30^. Soil net P-mineralization flux estimated based using this method is subject to uncertainty because it does not include contributions from plant roots that could potentially affect the C input and P exchange in the PVC pipes. However, the net soil P mineralization flux was corroborated by estimates from other measurements that integrate all plant and microbial processes, namely microbial P, phosphatase enzyme, available P concentrations and soil P concentrations measured using the Hedley fractionation method. To estimate net P-mineralization fluxes in deeper soil layers (10–30 cm, 30–60 cm), we assumed that the net mineralization activity was proportional to organic matter content, microbial biomass and fine-root biomass, and applied the proportion of change of measured soil C, microbial C and fine-root C across depth for each plot to the 0–10 cm measured net P-mineralization flux. The values obtained with the three variables were very similar, differing by 4.5%; we therefore report values estimated using soil C only. The soil P-leaching flux was estimated based on phosphate concentration collected in deeper soils (35–75 cm) using a water suction lysimeter^30^, assuming a water efflux of 20 ml m^−2^ d^−1^ through drainage at the site. The atmospheric P-deposition flux at the site was extracted from a gridded dataset^78^.

### Statistical analyses

We calculated treatment averages and their s.d. based on the plot-level data (*n* = 3). We calculated the s.d. for the aggregated pools and fluxes (for example, total plant P pool) by summing the individual components that constitute the aggregated pool and flux for each plot and computing the s.d. within each treatment (*n* = 3). The CO_2_ treatment effect was calculated as the net difference between eCO_2_ and aCO_2_ plots, with its s.d. (SD_eff_) calculated by pooling the s.d. values of the aCO_2_ and eCO_2_ treatments (SD_amb_ and SD_ele_, respectively) as follows:

$${{\rm{SD}}}_{{\rm{eff}}}=\sqrt{\frac{{{\rm{SD}}}_{{\rm{amb}}}^{2}+\,{{\rm{SD}}}_{{\rm{ele}}}^{2}}{2}}$$

Owing to long-term environmental fluctuation and spatial heterogeneity across treatment plots and the limited number of replication in large-scale field-based experiment^5,17,20,79^, the classic dichotomous approach of statistical test based on *P* value alone may underestimate the more subtle responses in manipulative experiments such as EucFACE. We therefore used multiple analytical approaches to robustly quantify and interpret the CO_2_ responses, including using confidence intervals to indicate the effect size^80,81^ (Fig. 4 and Extended Data Figs. 4 and 5), using linear mixed-effect models to report statistical results^82^ (Supplementary Information 2.1), and using bootstrap resampling as a sensitivity test^83^ (Extended Data Figs. 7 and 8, Extended Data Table 1 and Supplementary Information 2.2).

Reporting the means and confidence intervals is a useful way of assessing uncertainties in data, which has been shown to be more effective to assess the relationships within data than the use of *P* values alone, regardless of the statistical significance^80,81^. We calculated the confidence interval for the CO_2_ effect size (CI_eff_) as:

$${{\rm{CI}}}_{{\rm{eff}}}={t}_{95}{{\rm{SD}}}_{{\rm{eff}}}\sqrt{\frac{1}{{n}_{1}}+\frac{1}{{n}_{2}}}$$

Where *t*_95_ is the critical value of the *t-*distribution at 95% with (*n*_1_ + *n*_2_−2) d.f., and *n*_1_ = *n*_2_ = 3 is the sample size for each CO_2_ treatment. Taking the same approach, we also calculated the confidence intervals at 85% and 75%, respectively, to demonstrate the decreasing level of confidence in the reported CO_2_ effect size. For the mean CO_2_ effect size to be statistically significant from the null hypothesis at the 95%, 85% and 75% confidence levels, the corresponding confidence intervals must not overlap with zero.

To investigate the main CO_2_ effect statistically and how temporal fluctuation may have affected the CO_2_ effect (or the lack thereof), we built a linear mixed-effect model with CO_2_ treatment, year and their interaction as fixed factors and treatment plot as a random factor. We followed the conventional approach to interpret these results (that is, *P*-value cut-off < 0.05 as an indication for statistical significance between the ambient and elevated CO_2_ treatment plots). The results of the linear mixed-effect models indicate a generally consistent main CO_2_ effect across time (Supplementary Information 2.1). We therefore reported only the main CO_2_ effect based on the time-averaged plot-level data in the main text, and took an evidence-based approach^84^ to interpret the statistical significance of these results.

**Extended Data Fig. 6:**
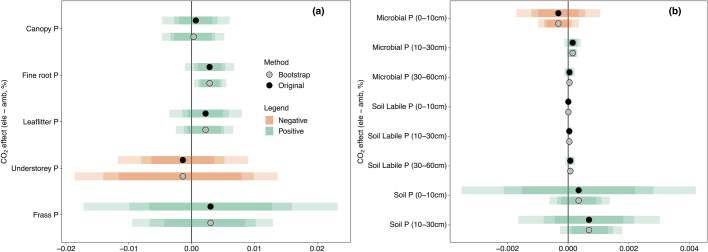
Comparison of the CO_2_ effect size (%) and the associated confidence intervals for major (a) plant and (b) soil phosphorus concentration variables, calculated by the original approach as reported in the main text (Original) and a bootstrapping method (Bootstrap, Supplementary Information 2.2). (**a**) The CO_2_ effects on plant P concentrations in canopy, fine root, leaf litter, understorey and frass P; (**b**) The CO_2_ effects on soil phosphorus concentrations of microbial P, labile soil P and total soil P at different soil depths (0–10 cm, 10–30 cm or 30–60 cm). Values indicate the mean absolute CO_2_ effect, calculated by using elevated minus ambient CO_2_ treatment (n = 3), with the coloured bars indicating confidence intervals at 95%, 85% and 75% (two-tailed t-test, with lighter colours indicating higher confidence levels).

**Extended Data Fig. 7:**
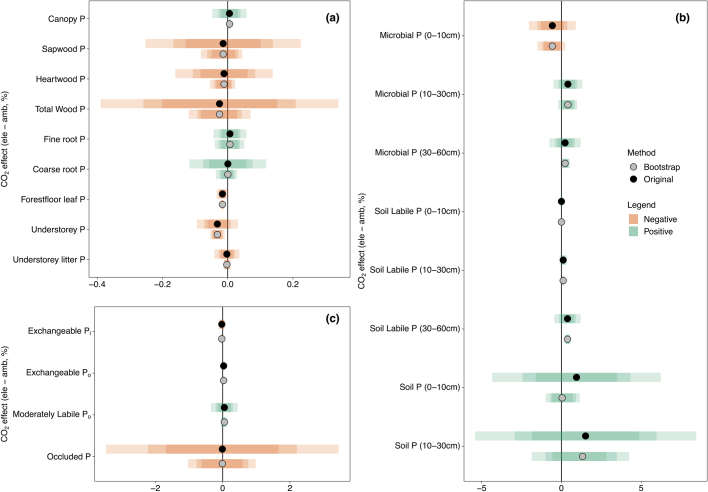
Comparison of the CO_2_ effect size (%) and the associated confidence intervals for major (a) plant (b) soil, and (c) operationally-defined soil phosphorus (P) pool variables, calculated by the original approach as reported in the main text (Original) and a bootstrapping method (Bootstrap, Supplementary Information 2.2). (**a**) The CO_2_ effects on plant P pools, including canopy, sapwood, heartwood, total wood, fine root, coarse root, forest floor leaf litter, understorey and, understorey litter; (**b**) the CO_2_ effects on soil P pools, including microbes, labile soil P and total soil P pools in the 0–10 cm, 10–30 cm, and 30–60 cm depths, respectively; (**c**) the CO_2_ effect on soil P pools in the top 10 cm of the soil, classified based on the Hedley fractionation method, including exchangeable inorganic P (P_i_), exchangeable organic P (P_o_), moderately labile P_o_, and occluded P, which is the remaining P that we consider to be relatively unavailable to plants. Values indicate the mean absolute CO_2_ effect, calculated by using elevated minus ambient CO_2_ treatment (n = 3), with the coloured bars indicating confidence intervals at 95%, 85% and 75% (two-tailed t-test, with lighter colours indicating higher confidence levels).

**Extended Data Fig. 8:**
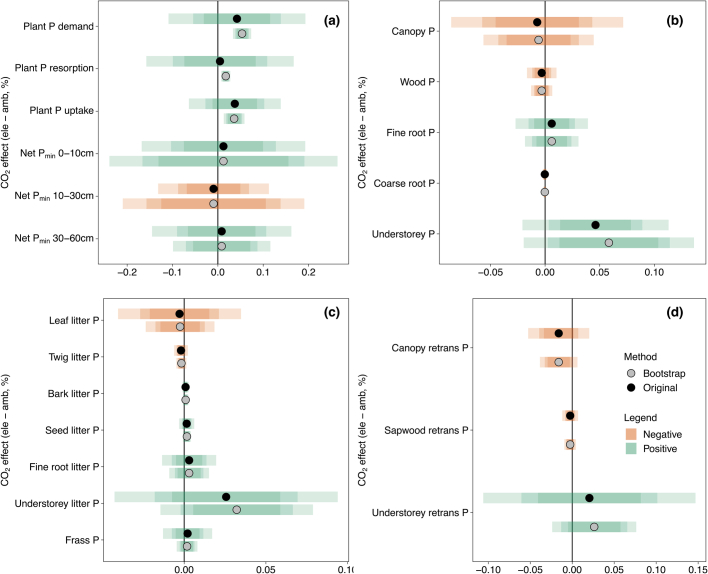
Comparison of the CO_2_ effect size (%) and the associated confidence intervals for major plant phosphorus (P) flux variables, calculated by the original approach as reported in the main text (Original) and a bootstrapping method (Bootstrap, Supplementary Information 2.2). (**a**) The CO_2_ effect on major plant and soil P fluxes, namely the CO_2_ effect on plant P demand, plant P resorption, plant P uptake and net soil P mineralization (Pmin) fluxes in the 0–10 cm, 10–30 cm and 30–60 cm depths, respectively; (**b**) The CO_2_ effect of canopy P, wood P, fine root P, coarse root P and understorey P fluxes; (**c**) The CO_2_ effect of leaf litter P fluxes for leaves, twigs, bark, seeds, understorey, fine root and frass; (**d**) the CO_2_ effect on retranslocation P fluxes for canopy, sapwood and understorey components. Values indicate the mean absolute CO_2_ effect, calculated by using elevated minus ambient CO_2_ treatment (n = 3), with the coloured bars indicating confidence intervals at 95%, 85% and 75% (two-tailed t-test, with lighter colours indicating higher confidence levels). Retrans stands for retranslocation flux; net P_min_ indicates net P mineralization flux.

Moreover, to quantify the uncertainties associated with temporal fluctuations in the measurements, we developed a bootstrapping method by randomly resampling datapoints from each CO_2_ treatment 1,000 times without ignoring the temporal fluctuation in the measurements. This approach can be considered as a sensitivity test. We then estimated the 95%, 85% and 75% confidence intervals of the bootstrapped CO_2_ effect based on the resampled data^83^. Results of this analysis suggest that the uncertainties associated with temporal fluctuations in the data do not affect the findings described in the main text (Extended Data Figs. 6–8 and Supplementary Information 2.2).

### Reporting summary

Further information on research design is available in the Nature Portfolio Reporting Summary linked to this article.

## Online content

Any methods, additional references, Nature Portfolio reporting summaries, source data, extended data, supplementary information, acknowledgements, peer review information; details of author contributions and competing interests; and statements of data and code availability are available at 10.1038/s41586-024-07491-0.

### Supplementary information

Supplementary Information Supplementary Information 1–4, including Supplementary Tables 1–5 and Supplementary Figs. 1 and 2.

Reporting Summary

## Figures and Tables

**Extended Data Table 1:**
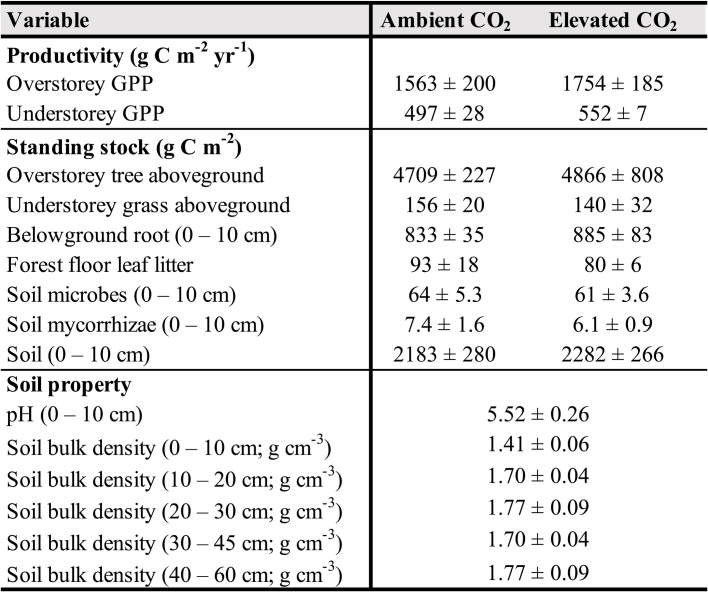
Key vegetation and soil variables estimated for EucFACE

GPP denotes gross primary production (g C m^−2^ yr^−1^). Values are treatment means ± standard deviations (n = 3).

## Data Availability

Data of this study are available at Figshare (10.6084/m9.figshare.25596213.v3)^85^.

## Code availability

The R scripts for analysing the data are available at GitHub (https://github.com/mingkaijiang/EucFACE_Phosphorus_Budget_Paper.git).
