# Supplementary material for: Microbial competition for phosphorus limits the CO2 response of a mature forest
Source: Nature. 2024 Jun 5;630(8017):660–5. doi: 10.1038/s41586-024-07491-0 (PMC11186757; doi:10.1038/s41586-024-07491-0)
Supplement: Supplementary file 1 — Supplementary Information 1–4, including Supplementary Tables 1–5 and Supplementary Figs. 1 and 2. [file 41586_2024_7491_MOESM1_ESM.pdf]

nature portfolio

https://doi.org/10.1038/s41586-024-07491-0

Supplementary information

Microbial competition for phosphorus limits the CO_2 response of a mature forest

In the format provided by the authors and unedited

Supplementary Information

Microbial competition for phosphorus limits CO_2 response of a mature forest

Authors: Mingkai Jiang[1,2]†, Kristine Y. Crous[2]†*, Yolima Carrillo[2], Catriona A. Macdonald[2], Ian C. Anderson[2], Matthias M. Boer[2], Mark Farrell[3], Andrew N. Gherlenda[2], Laura Castañeda-Gómez[2,4], Shun Hasegawa[2,5], Klaus Jarosch[6,7], Paul J. Milham[2], Rául Ochoa-Hueso[8,9], Varsha Pathare[2,10], Johanna Pihlblad[2,11,12], Juan Piñeiro[2,13], Jeff R. Powell[2], Sally A. Power[2], Peter B. Reich[2,14,15], Markus Riegler[2], Sönke Zaehle[16], Benjamin Smith[2], Belinda E. Medlyn[2], David S. Ellsworth[2]

Affiliations

1. College of Life Sciences, Zhejiang University, Hangzhou, China, 310030

2. Hawkesbury Institute for the Environment, Western Sydney University, Locked Bag 1797, Penrith, 2751, Australia

3. CSIRO Agriculture and Food, Kaurna Country, Locked Bag 2, Glen Osmond, SA, 5064, Australia

4. SouthPole Environmental Services, Zurich, Switzerland

5. Department of Forestry and Forest Resources, Norwegian Institute of Bioeconomy Research (NIBIO), Ås, Norway

6. Institute of Geography, University of Bern, Bern, Switzerland

7. Agroecology and Environment, Agroscope, Zurich-Reckenholz, Switzerland

8. Department of Biology, IVAGRO, University of Cádiz, Campus de Excelencia Internacional Agroalimentario (CeiA3), Campus del Rio San Pedro, 11510 Puerto Real, Cádiz, Spain

9. Department of Terrestrial Ecology, Netherlands Institute of Ecology (NIOO-KNAW), P.O. Box 50, 6700 AB, Wageningen, the Netherlands

10. Institute of Genomic Biology, University of Illinois at Urbana-Champaign, Illinois 61801, USA

11. Birmingham Institute for Forest Research, University of Birmingham, Edgbaston B15 2TT, United Kingdom

12. School of Geography, University of Birmingham, Edgbaston B15 2TT, United Kingdom

13. ETSI Montes, Forestal y del Medio Natural, Universidad Politécnica de Madrid, Ciudad Universitaria s/n, 28040 Madrid, Spain

14. Department of Forest Resources, University of Minnesota, St. Paul, MN 55108, USA.

15. Institute for Global Change Biology, and School for the Environment and Sustainability, University of Michigan, Ann Arbor, MI, 48109, USA

16. Max Planck Institute for Biogeochemistry, Hans-Knöll-Str. 10, 07745 Jena, Germany

*Corresponding author. Kristine Y. Crous Email: k.crous@westernsydney.edu.au

†These authors contributed equally to this work.

Table of contents

1. Additional data to place EucFACE in a global context……………………………………P1

2. Uncertainties associated with temporal fluctuations in data………………………………....P2

2.1. Linear mixed-effect model to quantify CO_2 by time interaction…………………………..P2

2.2. Bootstrapping approach to systematically quantify the uncertainties……………...……...P9

3. The CO_2 effect on the relative abundance of saprotrophic and mycorrhizal fungi in soils at EucFACE…………………………………………………………………………….…...P11

4. References cited……...……...……...……...……...……...……...……...……...……...……P13

Supplementary Table List

Supplementary Table 1. Phosphorus (P)-resorption coefficient calculated for canopy leaf, understorey shoot, and sapwood, under ambient and elevated CO_2…………...……………......P1

Supplementary Table 2. Statistical summary of the linear mixed-effects model on major phosphorus (P) concentration variables.…...…...…...…...…...…...…...…...…...…...…...…...P2

Supplementary Table 3. Statistical summary of the linear mixed-effects model on major phosphorus (P) pool variables.…...…...…...…...…...…...…...…...…...…...…...…...…...P4

Supplementary Table 4. Statistical summary of the linear mixed-effects model on major phosphorus (P) flux variables.…...…...…...…...…...…...…...…...…...…...…...…...…...P5

Supplementary Table 5. Relative abundances of ectomycorrhizal and saprotrophic fungi in soils at EucFACE during the months prior to CO_2 fertilization (sampling dates in 2012) and the first five years of CO_2 fertilization.…………...…………...…………...…………...…………......P11

Supplementary Table List

Supplementary Figure 1. Temporal fluctuations in the canopy P pool under ambient and elevated CO_2 treatment.…………. ..…………….... ..…………….... ..…………….... ..……....…………......P8

Supplementary Figure 2. Temporal fluctuations in fineroot P concentration under ambient and elevated CO_2 treatment.…...…...…... ... ...…...…...…...…...…...…...…...…...P9

1. Additional data to place EucFACE in a global context

The size of phosphorus (P) pool in stored in plants at EucFACE is relatively low, but it is broadly comparable to those estimated for broadleaf forests in the temperate and tropical or subtropical regions, in that it is above the 25th percentile of a regional dataset [1] (n=1969; Extended Data Figure 1d). Similarly, the amount of P stored in plant relative to soil at EucFACE (i.e., plant over soil P pool ratio) is broadly comparable to those estimated for tropical or subtropical broadleaf forest (Extended Data Figure 1e). This result suggests that the size of P pools estimated for EucFACE is representative of a large variety of forests.

In terms of the amount of P stored in soil microbes relative to the size of soil organic P pool, it is observed that EucFACE is on the relatively high end of the global estimate [2] (n=144; Extended Data Figure 1f). Soil microbes at EucFACE stored about 6.0 ± 1.43 g P m^{-2} over the top 60 cm of the soil, which accounts for 24% of the soil organic P pool. The global dataset indicates a mean fraction of 11.6% and a median fraction of 7.2%, significantly lower than the fraction estimated for EucFACE. This result suggests that microbes at EucFACE may have contained a disproportionally large amount of P in their biomass.

Plant P resorption played an important role to meet the annual plant nutrient demand. At EucFACE, canopy leaves on average retranslocated 55% of P from its tissue before leaf senescence (Supplementary Table 1). This canopy P resorption fraction is above the global average of 48% reported for evergreen broadleaf forests [3]. In comparison, understorey leaves are less efficient in resorbing P (43%) than the overstorey tree leaves. Sapwood is the most efficient plant organ in resorbing P, in that about 71% of P is retranslocated each year.

Supplementary Table 1. Phosphorus (P)-resorption coefficient calculated for canopy leaf, understorey shoot, and sapwood, under ambient and elevated CO_2. Canopy and understorey P resorption was calculated based on the difference between P concentrations in mature green leaves and leaf litter. Sapwood P resorption was calculated as the difference between sapwood P concentration and heartwood P concentration. Values are time-averaged means ± standard deviations (n=3).

Treatment	Canopy	Understorey shoot	Sapwood
aCO_2	0.546 ± 0.058	0.423 ± 0.110	0.710 ± 0.061
eCO_2	0.552 ± 0.060	0.417 ± 0.188	0.673 ± 0.055

2. Uncertainties associated with temporal fluctuations in data

2.1. Linear mixed effect model to quantify CO_2 by time interaction

In this study, we used data collected over a 6-year period (i.e., 2013-2018) to understand how atmospheric CO_2 enrichment (eCO_2) affects ecosystem P-cycle processes at EucFACE. There are three treatment plots for each CO_2 treatment (n=3), and data were collected in sub-replicates within each treatment plot at each sampling date. We calculated the means for each treatment plot to ensure the true replicate number is three for each CO_2 treatment at each sampling time point. In the overall budget reported in the main text, we reported the time-averaged values for each CO_2 treatment. To understand how temporal fluctuation may have affected the main CO_2 effect, we built a linear mixed-effects model, with CO_2 treatment and Date considered as fixed factors, and Plot considered as a random factor. We used R package "lme4" [4] to perform this analysis. The statistical results are summarized in Supplementary Tables 2, 3 and 4, for major P concentration, pool and flux variables, respectively.

As the statistics show, the Date factor is typically a strong predictor of the response variables (i.e., $P < 0.05$), but there is a general lack of main CO_2 effect, and Date typically does not interact with the Treatment factor (i.e., $P > 0.05$). This result indicates that the main CO_2 effect is consistent regardless of the temporal fluctuation of the data. The two exceptions are canopy P pool and fine root P concentration variables, both showing a statistically significant ($p < 0.05$) Treatment by Date interaction (Supplementary Table 2 and 3). More specifically, canopy P pool is higher (but statistically non-significant) in the eCO_2 treatment in all years except the later half of 2016 (Supplementary Figure 1a). In comparison, fine root P concentration is consistently higher (non-significant) in the eCO_2 treatment across all sampling dates except June and December in 2014 (Supplementary Figure 2a). However, when averaging these two pools at the annual timestep (Supplementary Figures 1b and 2b), none of the CO_2 comparisons show a significant treatment effect. Taken together, our statistical analysis suggests that the reported main CO_2 effect is generally consistent at different temporal scales.

Supplementary Table 2. Statistical summary of the linear mixed-effects model on major phosphorus (P) concentration variables. Year indicates the main effect of year, Treatment indicates the main effect of CO_2 treatment, whereas the Year: Treatment indicates the CO_2 by year interaction. Df and Df.res indicate the numerator and denominator degree of freedom, respectively.

Canopy P concentration	F	Df	Df.res	P-value
Year	11.09	5	67.55	<0.0001
Treatment	0.20	1	3.66	0.683
Year: Treatment	0.75	5	67.53	0.587
**Fine root P concentration**	**F**	**Df**	**Df.res**	**P-value**
Year	9.50	6	24	<0.0001
Treatment	4.15	1	4	0.111
Year: Treatment	3.20	6	24	0.019
**Leaf litter P concentration**	**F**	**Df**	**Df.res**	**P-value**
Year	4.20	5	20	0.009
Treatment	1.14	1	4	0.345
Year: Treatment	1.44	5	20	0.252
**Understorey P concentration**	**F**	**Df**	**Df.res**	**P-value**
Year	3.06	6	5.2	0.117
Treatment	0.28	1	3.3	0.632
Year: Treatment	0.02	2	4.7	0.985
**Frass P concentration**	**F**	**Df**	**Df.res**	**P-value**
Year	19.39	23	92	<0.0001
Treatment	0.17	1	4	0.700
Year: Treatment	0.96	23	92	0.771
**Microbial P concentration**	**F**	**Df**	**Df.res**	**P-value**
Year	71.45	6	24	<0.0001
Treatment	0.42	1	4	0.553
Year: Treatment	1.74	6	24	0.154
**Soil P concentration**	**F**	**Df**	**Df.res**	**P-value**
Year	2.76	5	20	0.047
Treatment	0.06	1	4	0.815
Year: Treatment	0.67	5	20	0.651
**Soil labile P concentration**	**F**	**Df**	**Df.res**	**P-value**
Year	46.58	15	60	<0.0001

**Treatment**	0.10	1	4	0.765
**Year: Treatment**	1.20	15	60	0.298

Supplementary Table 3. Statistical summary of the linear mixed-effects model on major phosphorus (P) pool variables. Year indicates the main effect of year, Treatment indicates the main effect of CO_2 treatment, whereas the Year: Treatment indicates the CO_2 by year interaction.

**Canopy P pool**	**F**	**Df**	**Df.res**	**P-value**
**Year**	22.90	399	1596	<0.0001
**Treatment**	0.08	1	4	0.786
**Year: Treatment**	1.54	399	1596	<0.0001
**Wood P pool**	**F**	**Df**	**Df.res**	**P-value**
**Year**	19.15	4	16	<0.0001
**Treatment**	0.04	1	4	0.858
**Year: Treatment**	0.28	4	16	0.888
**Fine root P pool**	**F**	**Df**	**Df.res**	**P-value**
**Year**	6.14	6	24	0.0005
**Treatment**	0.15	1	4	0.715
**Year: Treatment**	0.89	6	24	0.519
**Leaf litter P pool**	**F**	**Df**	**Df.res**	**P-value**
**Year**	4.43	45	180	<0.0001
**Treatment**	6.30	1	4	0.066
**Year: Treatment**	0.74	45	180	0.879
**Understorey P pool**	**F**	**Df**	**Df.res**	**P-value**
**Year**	8.54	17	68	<0.0001
**Treatment**	1.95	1	4	0.235
**Year: Treatment**	0.85	17	68	0.635
**Coarse root P pool**	**F**	**Df**	**Df.res**	**P-value**
**Year**	19.88	4	16	<0.0001
**Treatment**	0.0002	1	4	0.990

**Year: Treatment**	0.29	4	16	0.882
**Microbial P pool**	**F**	**Df**	**Df.res**	**P-value**
**Year**	77.77	6	24	<0.0001
**Treatment**	1.17	1	4	0.341
**Year: Treatment**	2.21	6	24	0.078
**Soil P pool**	**F**	**Df**	**Df.res**	**P-value**
**Year**	2.78	5	20	0.046
**Treatment**	0.001	1	4	0.974
**Year: Treatment**	0.63	5	20	0.681
**Soil labile P pool**	**F**	**Df**	**Df.res**	**P-value**
**Year**	46.28	15	60	<0.0001
**Treatment**	0.006	1	4	0.944
**Year: Treatment**	1.45	15	60	0.154

Supplementary Table 4. Statistical summary of the linear mixed-effects model on major phosphorus (P) flux variables. Year indicates the main effect of year, Treatment indicates the main effect of CO_2 treatment, whereas the Year: Treatment indicates the CO_2 by year interaction.

**Canopy P flux**	**F**	**Df**	**Df.res**	**P-value**
**Year**	18.82	48	192	<0.0001
**Treatment**	0.04	1	4	0.858
**Year: Treatment**	1.22	48	192	0.173
**Wood P flux**	**F**	**Df**	**Df.res**	**P-value**
**Year**	3.40	3	12	0.054
**Treatment**	0.41	1	4	0.558
**Year: Treatment**	0.71	3	12	0.562
**Fine root P flux**	**F**	**Df**	**Df.res**	**P-value**
**Year**	20.72	2	8	0.001
**Treatment**	0.26	1	4	0.637
**Year: Treatment**	0.46	2	8	0.646

**Leaf litter P flux**	**F**	**Df**	**Df.res**	**P-value**
**Year**	17.83	48	192	<0.0001
**Treatment**	0.03	1	4	0.871
**Year: Treatment**	1.10	48	192	0.322
**Understorey P flux**	**F**	**Df**	**Df.res**	**P-value**
**Year**	9.81	3	12	0.001
**Treatment**	4.36	1	4	0.105
**Year: Treatment**	1.19	3	12	0.355
**Coarse root P flux**	**F**	**Df**	**Df.res**	**P-value**
**Year**	3.30	3	12	0.058
**Treatment**	0.18	1	4	0.692
**Year: Treatment**	0.75	3	12	0.544
**Fine root litter P flux**	**F**	**Df**	**Df.res**	**P-value**
**Year**	20.72	2	8	0.001
**Treatment**	0.26	1	4	0.637
**Year: Treatment**	0.46	2	8	0.646
**Frass P flux**	**F**	**Df**	**Df.res**	**P-value**
**Year**	16.91	21	78	<0.0001
**Treatment**	0.08	1	4	0.796
**Year: Treatment**	0.59	21	78	0.911
**Understorey litter P flux**	**F**	**Df**	**Df.res**	**P-value**
**Year**	9.58	3	12	0.002
**Treatment**	1.63	1	4	0.271
**Year: Treatment**	1.22	3	12	0.344
**Canopy resorption P flux**	**F**	**Df**	**Df.res**	**P-value**
**Year**	19.71	48	192	<0.0001
**Treatment**	1.27	1	4	0.323
**Year: Treatment**	1.27	48	192	0.135
**Sapwood resorption P flux**	**F**	**Df**	**Df.res**	**P-value**
**Year**	3.30	3	12	0.058

**Treatment**	0.53	1	4	0.507
**Year: Treatment**	0.65	3	12	0.596
**Fine root resorption P flux**	**F**	**Df**	**Df.res**	**P-value**
**Year**	20.72	2	8	0.001
**Treatment**	0.26	1	4	0.637
**Year: Treatment**	0.46	2	8	0.646
**Coarse root resorption P flux**	**F**	**Df**	**Df.res**	**P-value**
**Year**	3.19	3	12	0.063
**Treatment**	0.27	1	4	0.631
**Year: Treatment**	0.69	3	12	0.575
**Understorey resorption P flux**	**F**	**Df**	**Df.res**	**P-value**
**Year**	7.26	3	12	0.005
**Treatment**	0.30	1	4	0.615
**Year: Treatment**	0.94	3	12	0.451
**Net P mineralization flux**	**F**	**Df**	**Df.res**	**P-value**
**Year**	8.55	13	52	<0.0001
**Treatment**	0.02	1	4	0.893
**Year: Treatment**	0.82	13	52	0.635
**Soil P leaching flux**	**F**	**Df**	**Df.res**	**P-value**
**Year**	14.69	11	34.4	<0.0001
**Treatment**	0.74	1	3.78	0.441
**Year: Treatment**	01.48	11	34.4	0.184

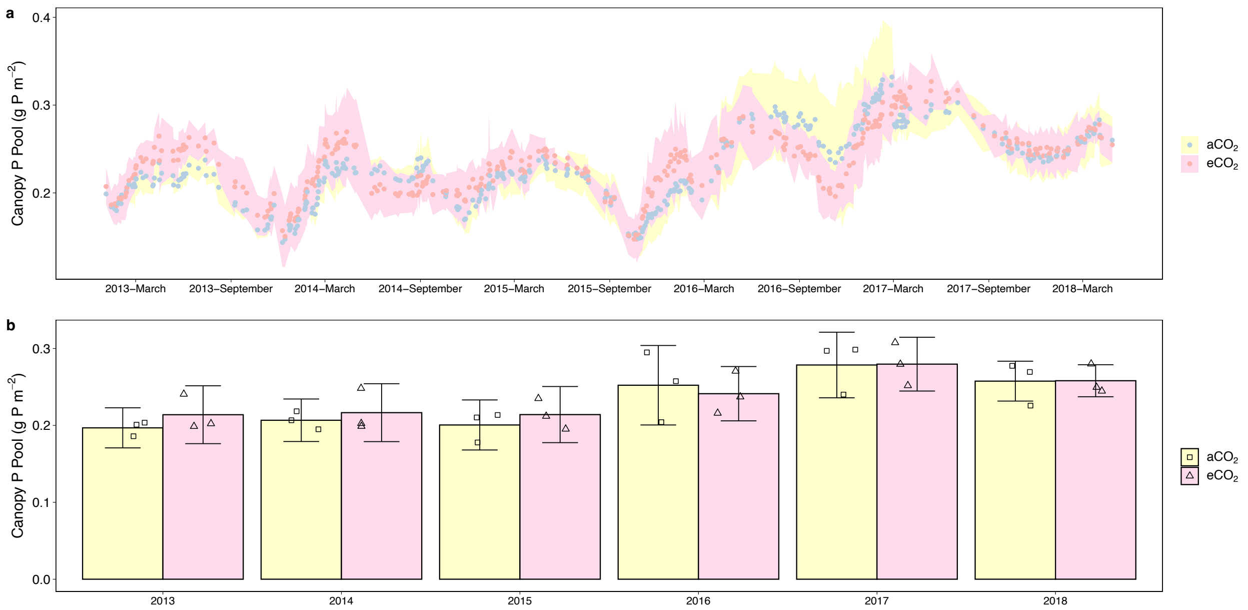

Supplementary Figure 1. Temporal fluctuations in the canopy P pool under ambient and elevated CO_2 treatment. Panel (a) shows the temporal fluctuations at each date, whereas panel (b) shows the annual-averaged P pools. The points and shaded areas in panel (a) indicates the mean ± standard deviation range, with dots indicating the treatment mean (n=3). Error Bars in panel (b) indicates the mean ± standard deviation range (n=3), and open squared and triangle dots denote plot-level data under ambient and elevated CO_2 treatment, respectively. Linear mixed-effects model indicates a lack of main CO_2 effect ($P < 0.05$) at the annual timestep.

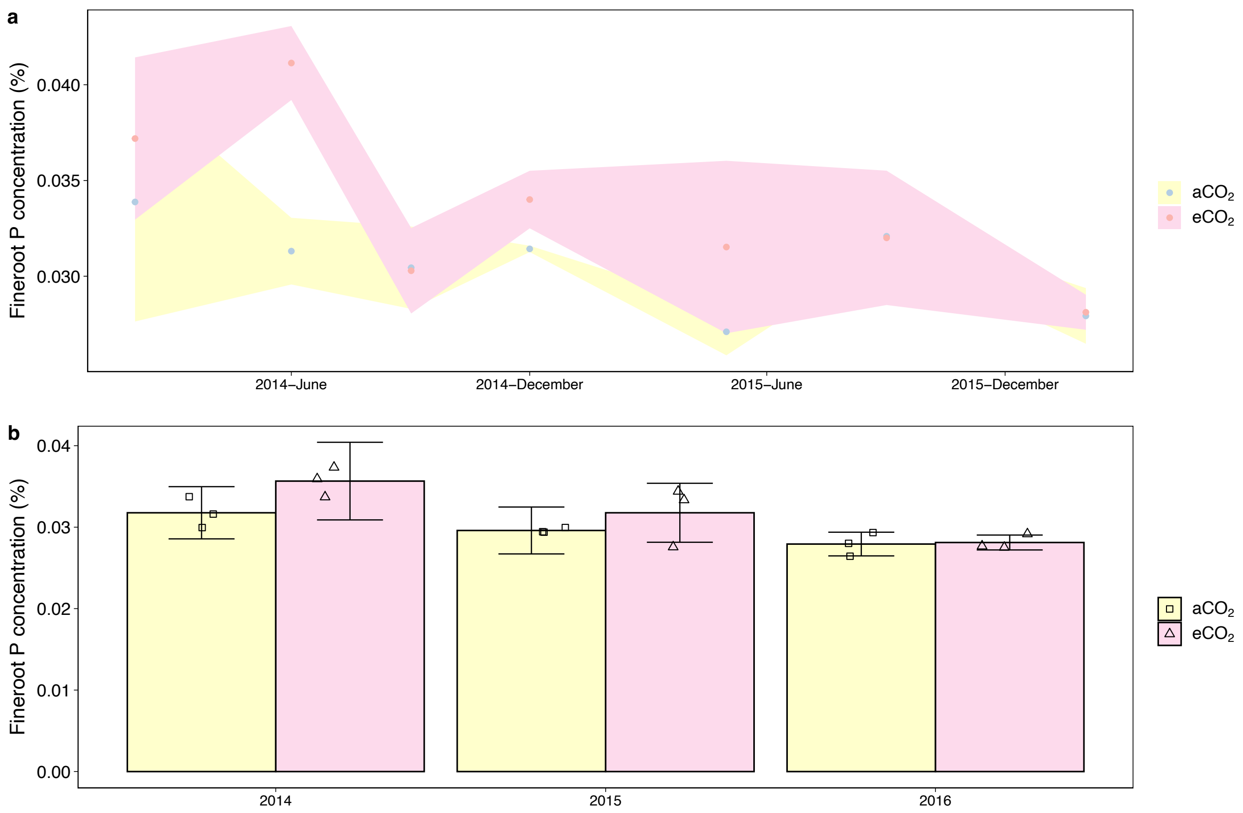

Supplementary Figure 2. Temporal fluctuations in fineroot P concentration under ambient and elevated CO_2 treatment. Panel (a) shows the temporal fluctuations at each date, whereas panel (b) shows the annual-averaged P pools. The points and shaded areas in panel (a) indicates the mean ± standard deviation range, with dots indicating the treatment mean (n=3). Error Bars in panel (b) indicates the mean ± standard deviation range (n=3), and open squared and triangle dots denote plot-level data under ambient and elevated CO_2 treatment, respectively. Linear mixed-effects model indicates a lack of main CO_2 effect ($P < 0.05$) at the annual timestep.

2.2. Bootstrapping approach to systematically quantify the uncertainties

We further developed a bootstrapping approach to quantify the uncertainty associated with the temporal fluctuation in data. For each variable that has a temporal data coverage, we calculated the plot-level mean at each sampling date, and then randomly selected three data points per treatment from the pool of sampling dates to calculate the treatment mean. We repeated this

process by 1000 times to obtain the bootstrapped mean and the associated confidence intervals. Essentially, this bootstrapping approach allows us to use different combinations of values to estimate the overall CO_2 effect and the uncertainties associated with the mean effect size. We used the R package "boot" [5,6] to perform this analysis. We then compared the estimated CO_2 effect size and the confidence intervals against those obtained from the original approach as reported in the main text.

Our results show that the CO_2 effect sizes and the associated confidence intervals reported in the main text are similar to those estimated based on the bootstrapping method (Extended Data Figures 6 – 8). For P concentration variables, the two approaches result in similar CO_2 effect sizes and similar confidence intervals (Extended Data Figure 6). For P pool variables, the bootstrapping method result in comparable confidence intervals relative to the original approached reported in the main text (Extended Data Figure 7). Similarly, for P flux variables, the bootstrapping method result in generally similar confidence intervals as compared to the original approached reported in the main text (Extended Data Figure 8). Notably, the sign of the CO_2 responses and the magnitude of the estimated effect sizes are comparable by the two approaches (Extended Data Figures 6 – 8). Therefore, we report the results based on the simple approach in the main text.

3. The CO_2 effect on the relative abundance of saprotrophic and mycorrhizal fungi in soils at EucFACE

Supplementary Table 5. Relative abundances of ectomycorrhizal and saprotrophic fungi in soils at EucFACE during the months prior to CO_2 fertilization (sampling dates in 2012) and the first five years of CO_2 fertilization. Estimated means represent log10-transformed response ratios (ectomycorrhizal: saprotrophic) of DNA sequence read counts following Illumina sequencing of the fungal ITS2 region; see Ref 7 for details of DNA extraction, sequencing and data processing methods. The soil samples were collected on each of the 17 dates indicated and, within 24 hours, passed through a 2mm sieve and stored at -80 °C until DNA extraction. Contrasts between ambient and elevated CO_2 conditions were estimated for each sampling date after fitting a linear mixed effects model that included nested random effects ('ring' [three per CO_2 treatment] and 'plot' [four per ring]; N=23-24 per sampling date, one sample from the first sampling date was removed due to a sequencing failure) and using Kenward-Roger degrees of freedom. The model indicated a non-significant effect of CO_2 treatment overall ($F = 0.78$; df = 1,4; $P = 0.43$), the only significant contrast was observed during the pre-treatment phase, and this contrast was only marginally significant ($P = 0.045$; consistent with the finding of a non-significant CO_2 treatment by date interaction: $F = 0.84$; df = 1,351.03; $P = 0.64$).

Date	Treatment	emmean	Contrast		
			Estimate	SE	P-value
2012-05-21	ambient	2.67	0.07	0.56	0.90
2012-05-21	elevated	2.60			
2012-09-03	ambient	2.47	1.15	0.55	0.04
2012-09-03	elevated	1.32			
2012-12-11	ambient	1.75	0.73	0.55	0.19
2012-12-11	elevated	1.02			
2013-03-11	ambient	2.17	0.70	0.55	0.21
2013-03-11	elevated	1.47			
2013-06-11	ambient	2.36	0.59	0.55	0.29
2013-06-11	elevated	1.77			
2013-09-03	ambient	2.63	0.63	0.55	0.26
2013-09-03	elevated	2.00			
2013-12-10	ambient	2.25	0.45	0.55	0.42
2013-12-10	elevated	1.79			
2014-03-10	ambient	1.92	-0.12	0.55	0.83
2014-03-10	elevated	2.04			
2014-06-17	ambient	0.77	0.09	0.55	0.87
2014-06-17	elevated	0.68			
2014-09-09	ambient	2.46	0.44	0.55	0.43
2014-09-09	elevated	2.02			

2014-11-17	ambient	2.65	0.08	0.55	0.89
2014-11-17	elevated	2.57			
2015-03-10	ambient	2.57	-0.51	0.55	0.36
2015-03-10	elevated	3.08			
2015-06-09	ambient	2.86	0.57	0.55	0.31
2015-06-09	elevated	2.29			
2015-09-07	ambient	2.78	0.18	0.55	0.74
2015-09-07	elevated	2.60			
2015-11-30	ambient	2.49	-0.24	0.55	0.66
2015-11-30	elevated	2.74			
2016-03-10	ambient	3.76	0.04	0.55	0.95
2016-03-10	elevated	3.72			
2017-08-21	ambient	2.95	-0.00	0.55	1.00
2017-08-21	elevated	2.95			

4. References cited

1. Z. Yu, G. Zhou, L. Liu, S. Manzoni, P. Ciais, D. Goll, J. Peñuelas, J. Sardans, W. Wang, J. Zhu, L. Li, J. Yan, J. Liu, X. Tang, Natural forests promote phosphorus retention in soil. *Global Change Biology.* **28**, 1678–1689 (2022).
2. X. Xu, P.E. Thornton, W.M. Post, A global analysis of soil microbial biomass carbon, nitrogen and phosphorus in terrestrial ecosystems. *Global Ecology and Biogeography*. **22**, 737-749 (2013).
3. D. Tian, J. Kattge, Y. Chen, W. Han, Y. Luo, J. He, H. Hu, Z. Tang, S. Ma, Z. Yan, Q. Lin, B. Schmid, J. Fang, A global database of paired leaf nitrogen and phosphorus concentrations of terrestrial plants. *Ecology*. **100** (2019), doi:10.1002/ecy.2812.
4. D. Bates, M. Mächler, B. Bolker, S. Walker, Fitting linear mixed-effects models using lme4. *Journal of Statistical Software*. **69**, 1:48 (2015).
5. A. Canty, B.D. Ripley, boot: Bootstrap R (S-Plus) Functions. R package version 1.3-28.1 (2022).
6. A.C. Davison, D.V. Hinkley, Bootstrap methods and their applications. Cambridge University Press, Cambridge, ISBN 0-521-57391-2 (1997).
7. L. Castañeda-Gómez, J.R. Powell, D.S. Ellsworth, E. Pendall, Y. Carrillo. The influence of roots on mycorrhizal fungi, saprotrophic microbes and carbon dynamics in a low-phosphorus Eucalyptus forest under elevated CO_2. *Functional Ecology*, **35**, 2056-2071 (2021).
