# Supplementary material for: Microbial competition for phosphorus limits the CO2 response of a mature forest
Source: Nature. 2024 Jun 5;630(8017):660–5. doi: 10.1038/s41586-024-07491-0 (PMC11186757; doi:10.1038/s41586-024-07491-0)
Supplement: Supplementary file 2 — Reporting Summary [file 41586_2024_7491_MOESM2_ESM.pdf]

nature portfolio

Corresponding author(s): Kristine Crous

Last updated by author(s): Apr 16, 2024

Reporting Summary

Nature Portfolio wishes to improve the reproducibility of the work that we publish. This form provides structure for consistency and transparency in reporting. For further information on Nature Portfolio policies, see our Editorial Policies and the Editorial Policy Checklist.

Statistics

For all statistical analyses, confirm that the following items are present in the figure legend, table legend, main text, or Methods section.

n/a	Confirmed	
☐	☒	The exact sample size (n) for each experimental group/condition, given as a discrete number and unit of measurement
☐	☒	A statement on whether measurements were taken from distinct samples or whether the same sample was measured repeatedly
☐	☒	The statistical test(s) used AND whether they are one- or two-sided<br>*Only common tests should be described solely by name; describe more complex techniques in the Methods section.*
☒	☐	A description of all covariates tested
☐	☒	A description of any assumptions or corrections, such as tests of normality and adjustment for multiple comparisons
☐	☒	A full description of the statistical parameters including central tendency (e.g. means) or other basic estimates (e.g. regression coefficient) AND variation (e.g. standard deviation) or associated estimates of uncertainty (e.g. confidence intervals)
☐	☒	For null hypothesis testing, the test statistic (e.g. F, t, r) with confidence intervals, effect sizes, degrees of freedom and P value noted<br>*Give P values as exact values whenever suitable.*
☒	☐	For Bayesian analysis, information on the choice of priors and Markov chain Monte Carlo settings
☐	☒	For hierarchical and complex designs, identification of the appropriate level for tests and full reporting of outcomes
☐	☒	Estimates of effect sizes (e.g. Cohen's d, Pearson's r), indicating how they were calculated

Our web collection on statistics for biologists contains articles on many of the points above.

Software and code

Policy information about availability of computer code

Data collection	Raw data was produced via chemical analyses, no software was used during data collection.
Data analysis	The R scripts for analyzing the data is available via GitHub (https://github.com/mingkaijiang/EucFACE_Phosphorus_Budget_Paper.git).

For manuscripts utilizing custom algorithms or software that are central to the research but not yet described in published literature, software must be made available to editors and reviewers. We strongly encourage code deposition in a community repository (e.g. GitHub). See the Nature Portfolio guidelines for submitting code & software for further information.

Data

Policy information about availability of data

All manuscripts must include a data availability statement. This statement should provide the following information, where applicable:

- Accession codes, unique identifiers, or web links for publicly available datasets
- A description of any restrictions on data availability
- For clinical datasets or third party data, please ensure that the statement adheres to our policy

Data Availability Statement: Data of this study can be accessed via the Figshare link (https://doi.org/10.6084/m9.figshare.25596213.v1).

Research involving human participants, their data, or biological material

Policy information about studies with human participants or human data. See also policy information about sex, gender (identity/presentation), and sexual orientation and race, ethnicity and racism.

Reporting on sex and gender	not applicable
Reporting on race, ethnicity, or other socially relevant groupings	not applicable
Population characteristics	not applicable
Recruitment	not applicable
Ethics oversight	not applicable

Note that full information on the approval of the study protocol must also be provided in the manuscript.

Field-specific reporting

Please select the one below that is the best fit for your research. If you are not sure, read the appropriate sections before making your selection.

☐ Life sciences ☐ Behavioural & social sciences ☒ Ecological, evolutionary & environmental sciences

For a reference copy of the document with all sections, see nature.com/documents/nr-reporting-summary-flat.pdf

Ecological, evolutionary & environmental sciences study design

All studies must disclose on these points even when the disclosure is negative.

Study description	The Eucalyptus Free-Air CO2 Enrichment (EucFACE) experiment is located in a remnant patch of native Cumberland Plain woodland on an ancient alluvial floodplain in western Sydney, Australia (33o 37'S, 150o 44'E, 30 m in elevation).
Research sample	A mature forest dominated by Eucalyptus tereticornis at an unmanaged site for over 90 years. The open woodland (600-1000 trees ha-1) is dominated by Eucalyptus tereticornis Sm. in the overstorey while the understorey is dominated by the C3 grass, Microlaena stipoides (Labill.) R.Br . The vegetation within three randomly-selected plots (~450 m2 each) has been exposed to an elevated CO2 treatment (eCO2) aiming for a CO2 mole fraction of 150 µmol mol-1 above ambient concentration since February 2013. The other three plots were used as control plots representing the ambient CO2 treatment (aCO2), with identical infrastructure and instrumentation as the treatment plots. Aboveground and belowground samples were collected in all plots.
Sampling strategy	By virtue of the experimental design, the samples size is n=3 for all sampling.<br>See methods for detailed sampling procedures of the different ecosystems components both above- and belowground.
Data collection	Most co-authors contributed to data collection as detailed in the 'author contribution statement'. Each author focused on a given component of the ecosystem for which P data was collected and then synthesized across years to a comprehensive budget of P cyclin in a mature forest.
Timing and spatial scale	Data was collected between 2013 and 2018, representing the first 6 years of CO2 treatment in the experiment. Most data streams were collected regularly (every few weeks to months), but it varied depending on the variables involved. See method section for specific details.
Data exclusions	No data relevant to building a phosphorus budget was excluded.
Reproducibility	All attempts to repeat the experiment were successful, all data analyses are reproducible via a github repository
Randomization	Allocations of CO2 treatment was random with sampling inside the plots also being randomized.
Blinding	The three CO2 fertilization treatment plots were assigned a random ID number during the experiment. For the chemical analyses, standards and blind standards were submitted and were within a few percent of the known value. We accepted chemical analysis values from data contributors according to their own quality control and blinding approaches.

Did the study involve field work? ☒ Yes ☐ No

Field work, collection and transport

Field conditions	The field site is characterized by a humid temperate-subtropical transitional climate with a mean annual temperature of 17 °C and mean annual precipitation of ~800 mm (1881 - 2014, Bureau of Meteorology, station 067105 in Richmond, NSW, Australia; http://www.bom.gov.au). The soil is formed from weakly-organized alluvial deposits and is primarily an Aeric Podosol with areas of Densic Podosol (Australian soil classification).
Location	Richmond, NSW, Australia
Access & import/export	not applicable, the experiment is located on a University campus, for which permits were not needed. Accessibility is assessed by University-employed site managers.
Disturbance	The forest had been undisturbed for over 90 years. Specific paths from and to the experimental plots are laid out to protect the soil environment and minimize ecosystem disturbance.

Reporting for specific materials, systems and methods

We require information from authors about some types of materials, experimental systems and methods used in many studies. Here, indicate whether each material, system or method listed is relevant to your study. If you are not sure if a list item applies to your research, read the appropriate section before selecting a response.

Materials & experimental systems

n/a	Involved in the study	
☒	☐	Antibodies
☒	☐	Eukaryotic cell lines
☒	☐	Palaeontology and archaeology
☒	☐	Animals and other organisms
☒	☐	Clinical data
☒	☐	Dual use research of concern
☐	☒	Plants

Methods

n/a	Involved in the study	
☒	☐	ChIP-seq
☒	☐	Flow cytometry
☒	☐	MRI-based neuroimaging

Dual use research of concern

Policy information about dual use research of concern

Hazards

Could the accidental, deliberate or reckless misuse of agents or technologies generated in the work, or the application of information presented in the manuscript, pose a threat to:

No	Yes	
☒	☐	Public health
☒	☐	National security
☒	☐	Crops and/or livestock
☒	☐	Ecosystems
☒	☐	Any other significant area

Experiments of concern

Does the work involve any of these experiments of concern:

No	Yes	
☒	☐	Demonstrate how to render a vaccine ineffective
☒	☐	Confer resistance to therapeutically useful antibiotics or antiviral agents
☒	☐	Enhance the virulence of a pathogen or render a nonpathogen virulent
☒	☐	Increase transmissibility of a pathogen
☒	☐	Alter the host range of a pathogen
☒	☐	Enable evasion of diagnostic/detection modalities
☒	☐	Enable the weaponization of a biological agent or toxin
☒	☐	Any other potentially harmful combination of experiments and agents

Plants

Seed stocks	An already established mature Eucalyptus woodland was used in the study, undisturbed for almost 100 years, no seed stocks used
Novel plant genotypes	Not applicable, this was a field study using the genotypes provided by nature.
Authentication	not applicable
